# Genome-environment associations along elevation gradients in two snowbed species of the North-Eastern Calcareous Alps

**DOI:** 10.1186/s12870-023-04187-x

**Published:** 2023-04-19

**Authors:** Sabine Felkel, Karin Tremetsberger, Dietmar Moser, Juliane C. Dohm, Heinz Himmelbauer, Manuela Winkler

**Affiliations:** 1 Institute of Computational Biology, Department of Biotechnology, University of Natural Resources and Life Sciences, Vienna, Vienna, 1190 Austria; 2 Institute of Botany, Department of Integrative Biology and Biodiversity Research, University of Natural Resources and Life Sciences, Vienna, Vienna, 1180 Austria; 3 Biodiversity Dynamics and Conservation Group, Department of Botany and Biodiversity Research, University of Vienna, Vienna, 1030 Austria; 4 GLORIA Coordination, Institute for Interdisciplinary Mountain Research, Austrian Academy of Sciences, Vienna, 1190 Austria; 5 GLORIA Coordination, Institute of Botany, Department of Integrative Biology and Biodiversity Research, University of Natural Resources and Life Sciences, Vienna, Vienna, 1190 Austria

**Keywords:** Alpine plants, Climate change, Adaptation, Genetic structure, Gene flow, Isolation by distance, Environmental association analysis, *Achillea clusiana*, *Campanula pulla*, Genotyping-by-sequencing

## Abstract

**Background:**

Anthropogenic climate change leads to increasing temperatures and altered precipitation and snowmelt patterns, especially in alpine ecosystems. To understand species’ responses to climate change, assessment of genetic structure and diversity is crucial as the basis for the evaluation of migration patterns, genetic adaptation potential as well as the identification of adaptive alleles.

**Results:**

We studied genetic structure, diversity and genome-environment associations of two snowbed species endemic to the Eastern Alps with a large elevational range, *Achillea clusiana* Tausch and *Campanula pulla* L. Genotyping-by-sequencing was employed to assemble loci de novo, call variants and perform population genetic analyses. Populations of either species were distinguishable by mountain, and to some extent by elevation. We found evidence for gene flow between elevations. Results of genome-environment associations suggested similar selective pressures acting on both species, emanating mainly from precipitation and exposition rather than temperature.

**Conclusions:**

Given their genetic structure and amount of gene flow among populations the two study species are suitable to serve as a model for genetic monitoring of climate change adaptation along an elevation gradient. Consequences of climate change will predominantly manifest via changes in precipitation and, thus, duration of snow cover in the snowbeds and indirectly via shrub encroachment accompanied by increasing shading of snowbeds at lower range margins. Assembling genomes of the study species and studying larger sample sizes and time series will be necessary to functionally characterize and validate the herein identified genomic loci putatively involved in adaptive processes.

**Supplementary Information:**

The online version contains supplementary material available at 10.1186/s12870-023-04187-x.

## Background

Anthropogenic climate change will lead to a projected increase in global mean temperature of 1.4–4.4 °C compared to pre-industrial levels by the end of the century [1]. However, there will be large regional differences, with high latitudes and high elevations experiencing more pronounced temperature increases than the global average [1, 2]. For instance, temperature increase in the European Alps [3, 4] has been roughly double the global mean so far. In addition to direct effects, increase in temperature also affects precipitation and snowmelt patterns which in turn have pronounced effects on alpine ecosystems [5].

A wealth of studies and meta-analyses confirm that climate change is already affecting biota (e.g., [6–8]). Their responses can be classified into four categories: range shifts, phenotypic plasticity, (genetic) adaptation, or extinction [9, 10]. Ranges of many species representing a wide variety of taxonomic groups have shifted into the expected directions in a warming world: towards higher latitudes and higher elevations (e.g., [11–13]). Most notably, with a temperature increase of + 2.2 K an upwards shift of the tree line will lead to the loss of about a quarter of the lower alpine habitat area, and of more than half of the upper alpine/subnival habitat area [14], severely curtailing trailing edges of alpine plant species. Range shifts are also predicted for genetic groups within species, for instance, contact zones between cold- and warm-adapted genetic groups are projected to move to the north and east within the Alps [15]. Changes in local conditions can be buffered to a limited extent by phenotypic plasticity, which allows an organism to change its morphological, physiological or phenological phenotype in accordance with the environment without underlying changes in the DNA sequence [16].

Nevertheless, climate change can also trigger genetically based adaptive evolution, which may proceed rapidly (e.g., [17, 18]). The influence of climate change on the genetic constitution of populations may be straightforward, e.g., when a particular allele conferring fitness advantages through increased thermal tolerance is favored by selection under rising temperatures. However, adaptive genetic differentiation linked to temperature has only been identified for a restricted number of species (e.g., [19–21], reviewed in [22]) – and the spread of advantageous alleles under climate change is largely unknown [23].

To understand species responses to climate change, in particular genetic adaptation, it is essential to consider differences in the evolutionary ecology of edge versus central populations. Range limits may be caused by Allee effects, temporal or spatial variation in dispersal (e.g., due to dispersal barriers), biotic interactions, environmental gradients, and interactions between these factors (reviewed in [24]). Selection regimes may differ, with stabilizing selection dominating at the center and directional selection at the periphery of the range. Peripheral populations may respond to selective pressure by adapting to environments beyond range limits, given sufficient genetic variation [25, 26]. However, genetic diversity usually decreases from the center to the edges of the geographical range, although these differences are small [27]. The effect of gene flow from central to peripheral populations ranges from facilitation of local adaptation by providing the necessary genetic variation [28, 29] to its prevention due to the influx of alleles maladapted to the conditions at the periphery [30, 31]. The direction and strength of selection may be decisive which of these effects prevails [32, 33].

Besides monitoring over time, one approach to infer species’ responses to climate change [34] is based on observations of natural populations across space – *i.e.*, latitudinal [35] or elevation [36] gradients. Species responses to climate change are often hindered or restricted by coincidental land-use changes resulting in fragmentation and habitat destruction (e.g., [37]). Alpine ecosystem are an ideal system to study climate change effects, since human settlements are scarce at higher elevations where intensive agriculture is not feasible [38] and agriculture-related or other aspects of global change are less confounding.

Genetic structure and diversity of alpine species have also been shaped by past climatic fluctuations during and after the Pleistocene [39]. During cold phases of the glacial cycle species retreated to ice-free (mostly) peripheral refugia, from where the Alps were recolonized again after the retreat of the ice shield [40]. At the north-eastern edge of the Alps a series of peripheral glacial refugia for several calcicolous alpine species was located [40–43]. The three mountains studied here are located in the North-Eastern Calcareous Alps last glacial maximum (LGM) refugium of subalpine and alpine plants as defined by Schönswetter et al. (2005; [41]) and may therefore have served as glacial refugia. Isolation in refugia leads to genetic divergence and an accumulation of private alleles [39].

Here, we study genetic structure and diversity, as well as genome-environment associations, of two snowbed species endemic to the Eastern Alps with a large elevational range, *Achillea clusiana* Tausch and *Campanula pulla* L. Endemic species were chosen in order to be able to cover a substantial part of their distribution area with a limited number of populations. Snowbeds are patchily distributed within the alpine grassland and the subalpine *Pinus mugo* krummholz belt on sites with a long-lasting snow cover. This patchiness implies natural fragmentation of the species’ habitat, and population dynamics influenced by patch size and – to a lesser degree – connectivity and habitat suitability [44]. As the study area encompasses mountains of comparatively low elevations (max. elevation is 2,277 m a.s.l.), the study species already occupy available habitat patches up to the highest elevations. Thus, the leading upper range edge is curtailed, while snow-cover duration is projected to decrease with climate change, and consequently habitat connectivity will considerably decline in the study area [45].

The aim of our study was to assess the suitability of the two chosen species to serve as a model for genetic monitoring along an elevation gradient. Specifically, we address the following questions and hypotheses drawing on the elevational gradient as a space-for-time substitute [46]: (1) *Genetic structure, diversity and rarity:* Suitable habitats are discontinuously distributed along the elevational gradient for both species, so we expect genetic differentiation among populations. We also expect the highest genetic diversity in the uppermost populations, where suitable habitats are more abundant, and decreasing genetic diversity with decreasing elevation in both species. Additionally, we aim at quantifying the proportion of private alleles in the populations at the lower (trailing) range edge, which have the highest risk of disappearing due to shrub encroachment and upwards advancement of the tree line [14]. (2) *Gene flow* among mountains is expected to be limited and to follow an isolation-by-distance (IBD) pattern. Within a mountain, we expect seed dispersal mainly from the upper to the lower populations, following gravity and the flow of water. Upward gene flow, which would be necessary to keep pace with the changing climate, could be accomplished by upwinds or dispersal and/or pollination through animal vectors. (3) *Genome-environment associations:* We aim at identifying loci under selection correlated with temperature-related abiotic variables (i.e., downscaled temperature, elevation, orientation) and precipitation, which both are important determinants of snow depth.

## Results

### Sampling and environmental data

**Fig. 1:**
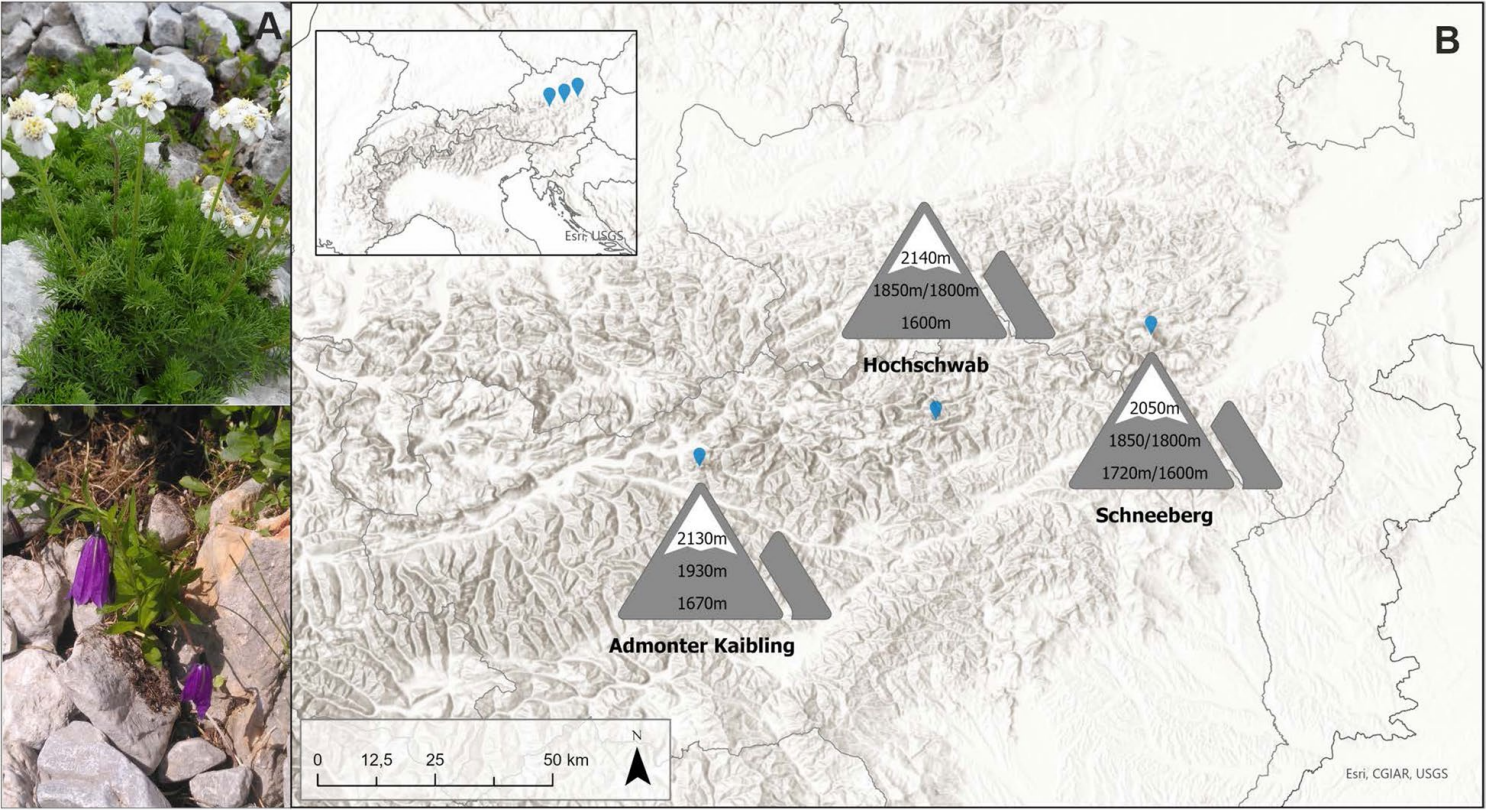
**A** Habitus of the study species *Achillea clusiana* (upper panel) and *Campanula pulla* (lower panel). **B** Sampling locations on three mountains in the Austrian North-Eastern Calcareous Alps. On each mountain, populations of the two species were sampled at three different elevations. In case of differences in elevations between the sampling locations of the two species, the elevation is given first for *A. clusiana* and then for *C. pulla*. Photos: Manuela Winkler

We employed reduced-representation genomic sequencing and assessed if such an approach is suitable to distinguish between localities of populations and to determine genetic divergence related to local adaptation. In this pilot study, two alpine plant species, *Achillea clusiana* and *Campanula pulla*, were sampled from three Austrian mountains located in the Northeastern Calcareous Alps, at three different elevations in July 2019. From each sampling site, five individuals were collected. The three mountains were Schneeberg (2,076 m a.s.l.), Lower Austria, Hochschwab (2,277 m a.s.l.), Styria, and the Admonter Kaibling (2,196 m a.s.l.), Styria. On each mountain three populations covering the elevation range of the two species were sampled, *i.e.*, at the "lower" (1,600–1,720 m), "medium" (1,800–1,930 m), and "upper" (2,050–2,140 m) part of their vertical distribution area. In total, 92 samples from 90 individuals were analyzed (Fig. 1, Table 1). Individuals of a given population were located in snowbeds or moist calcareous rock fissures and scree within a radius of < 50 m. GPS coordinates, elevation and exposition were recorded. Exposition recorded in degree was transformed into radiant and then into eastness (sin(α)) and northness (cos(α)), thus, creating two linear variables from -1, representing west for eastness and south for northness, to 1, representing east for eastness and north for northness, respectively. Precipitation, minimum, maximum, and mean temperature were downscaled from CHELSA data [47] to the specific sampling locations using the delta method [see Methods]. Annual precipitation ranged from 1316 mm in the lower Admonter Kaibling population to 1733 mm in the upper Hochschwab population, and mean annual temperature from -0.68 °C in the upper Hochschwab population to 2.29 °C in the lower Admonter Kaibling population, respectively (CHELSA data 1979–2013; Table 1, Table S1).

**Table 1:** Sampled populations of the alpine snowbed plant species *Achillea clusiana* and *Campanula pulla* in the Northeastern Calcareous Alps, Austria

Population^a^	Elevation [m a.s.l.]	Exposition [°]	N	E	MAT [°C]^b^	Precipitation [mm]^b^	Individual IDs	Herbarium accession No	NCBI BioSample accession
*Achillea clusiana*									
Admonter Kaibling (K)—L	1670	180	47.5461	14.5150	2.29	1316	A116–A120	WHB 78,444	SAMN28952182–SAMN28952186
Admonter Kaibling (K)—M	1930	205	47.5478	14.5184	0.78	1419	A121–A125	WHB 78,445	SAMN28952187–SAMN28952191
Admonter Kaibling (K)—U	2130	315	47.5488	14.5230	-0.34	1499	A126–A130	WHB 78,446	SAMN28952192–SAMN28952196
Hochschwab (H)—L	1600	30	47.6449	15.2638	2.21	1395	A101–A105	WHB 78,441	SAMN28952166–SAMN28952171
Hochschwab (H)—M	1850	225	47.6171	15.1601	0.93	1567	A106–A110	WHB 78,442	SAMN28952172–SAMN28952176
Hochschwab (H)—U	2140	20	47.6212	15.1471	-0.68	1733	A111–A115	WHB 78,443	SAMN28952177–SAMN28952181
Schneeberg (S)—L	1720	165	47.7845	15.8085	1.44	1369	A131–A135	WHB 78,447	SAMN28952197–SAMN28952201
Schneeberg (S)—M	1850	345	47.7780	15.8050	0.58	1439	A136–A140	WHB 78,448	SAMN28952202–SAMN28952206
Schneeberg (S)—U	2050	100	47.7698	15.8067	-0.37	1511	A141–A145	WHB 78,449	SAMN28952207–SAMN28952211
*Campanula pulla*									
Admonter Kaibling (K)—L	1670	180	47.5461	14.5150	2.29	1316	C116–C120	WHB 78,453	SAMN28952227–SAMN28952232
Admonter Kaibling (K)—M	1930	205	47.5478	14.5184	0.78	1419	C121–C125	WHB 78,454	SAMN28952233–SAMN28952237
Admonter Kaibling (K)—U	2130	315	47.5488	14.5230	-0.34	1499	C126–C130	WHB 78,455	SAMN28952238–SAMN28952242
Hochschwab (H)—L	1600	30	47.6450	15.2641	2.21	1395	C101–C105	WHB 78,450	SAMN28952212–SAMN28952216
Hochschwab (H)—M	1800	225	47.6168	15.1600	0.93	1567	C106–C110	WHB 78,451	SAMN28952217–SAMN28952221
Hochschwab (H)—U	2140	20	47.6212	15.1471	-0.68	1733	C111–C115	WHB 78,452	SAMN28952222–SAMN28952226
Schneeberg (S)—L	1600	5	47.7860	15.8088	1.80	1339	C131–C135	WHB 78,456	SAMN28952243–SAMN28952247
Schneeberg (S)—M	1800	345	47.7795	15.8066	0.82	1419	C136–C140	WHB 78,457	SAMN28952248–SAMN28952252
Schneeberg (S)—U	2050	100	47.7698	15.8067	-0.37	1511	C141–C145	WHB 78,458	SAMN28952253– SAMN28952257

Given are elevation, exposition, WGS 1984 coordinates (N, E), mean annual temperature (MAT), annual precipitation, individual IDs and accession number of the herbarium of the University of Natural Resources and Life Sciences, Vienna (WHB) and NCBI BioSample accessions

^a^*L* Population at lower elevational range limit, *M* Intermediate, *U* Upper elevational range limit

^b^downscaled from CHELSA data 1979–2013

### Data generation

For each of the samples we performed restriction site associated DNA sequencing (RAD-seq; [48]) in a modification of the genotyping-by-sequencing (GBS) protocol from [49] using the restriction enzyme *Ape*KI which performed best out of the three tested enzymes *Ape*KI, *Eco*T22I and *Pst*I, showing no cutting sites within prominent repeats, and generating many fragments. *Eco*T22I appeared to cut in repeats as indicated by strong spikes in the fragment trace especially in *A. clusiana*. *Pst*I, on the other hand, had smooth traces in both species, but sampled fewer sites in the genome than *Ape*KI.

A total of 511 million read pairs (2 × 150 nt) was generated by Illumina sequencing. Demultiplexing and filtering resulted in 8.1 million read pairs on average per sample (range: 3.3–13.8 million) with a mean of 7.1 million for *A. clusiana* and a mean of 9.0 million for *C. pulla*, respectively (filter4 in Table S2, Table S3, Fig. S1 and Fig. S2).

### Ploidy estimation

Intraspecific variation in ploidy occurs in a wide range of species and polyploidy can accelerate evolutionary adaptation by increasing the genetic diversity. Ploidy can be inferred directly by measuring DNA content or from read mappings to a reference genome. If inferred from sequencing data, ploidy levels can be distinguished based on distribution of base frequencies at biallelic variable sites that are characteristic for each ploidy level. *nQuire* [50] was used for ploidy estimation of each analyzed sample and the diploid model always had best fit between ideal and empirical histograms (see Methods for details). The results indicated that each of the sequenced samples most likely had a diploid genome, confirming previous reports of diploidy in both study species [51–53].

### Read clustering and variant calling

The assembly and variant calling steps were performed using the program Stacks [54] with sequencing reads trimmed to a length of 90 bases (filter5 in Table S3).

First, putative alleles were built for each sample separately by de novo aligning forward reads into exactly-matching stacks. By comparing the stacks, sets of putative loci were formed and variants were identified using a maximum likelihood framework [55]. On average, 136,275 loci with a mean coverage of twelve were obtained per *A. clusiana* sample and 117,558 loci with 19-fold mean coverage were obtained for each *C. pulla* sample, respectively (Fig. S3 and Table S4). The measured genome size of *A. clusiana* was ~ 2.7–2.8 pg/1C and that of *C. pulla* ~ 1.4–1.5 pg/1C (both from the upper Hochschwab population).

A set of consensus loci (locus catalog) for each species was built by comparing the combined sets of stacks from all samples of either species and merging alleles. While 1.53 million consensus loci were built for *A. clusiana*, only 0.66 million consensus loci were built for *C. pulla*. The relation of the size of the locus catalogs contrasted with the relation of input reads comprising 106 million for *A. clusiana* and 129 million for *C. pulla* (Fig. S2 and Table S4), and can be attributed to the different sizes of the two genomes, hence, a differing number of restriction sites per genome.

Thereafter, the sets of stacks (putative loci) were searched against the respective species locus catalog for each sample separately. Read-pair information was then integrated; paired-end reads were assembled into a contig and merged with the single-end locus. Inclusion of paired-read information led to an increase of the mean effective per-sample coverage to 17 in *A. clusiana* and 24 in *C. pulla*. Contigs of *A. clusiana* had an average length of 144 bp and those of *C. pulla* had an average length of 149 bp.

The information gathered by all previous steps was combined in one final step. Per species, by looking at one assembled contig (locus) at a time, reads from each individual in the dataset were aligned to that contig and each individual was genotyped at each identified variant. We considered only loci that were represented in all nine populations. We kept only polymorphic loci and required a locus to be covered by at least 80% of the samples of a population to be processed for that population and a minimum minor allele frequency of at least 5% to process a variant. When assembling contigs using *Stacks* it may happen that reverse-complementary contigs are retained, subsequently leading to duplicated variant calls. This was the case for around one third of the genotyped loci and variant calls. Homologous loci were thus identified with *BLAST* (> 90% identity) and duplicates were discarded. We further filtered for genotype quality and mapping depth (Fig. S4). These filtering steps resulted in 18,587 *A. clusiana* variants on 9,475 contigs and 29,755 *C. pulla* variants on 15,153 contigs for downstream analyses (Fig. S5).

On average, 1.9 variants were detected per contig per species. Considering each sampled population separately, i.e., the five individuals from different mountains and elevations, there were 11,923 variants in *A. clusiana* and 18,236 variants in *C. pulla*, respectively, on average per population (Table S5).

### Genetic diversity and rarity

**Fig. 2:**
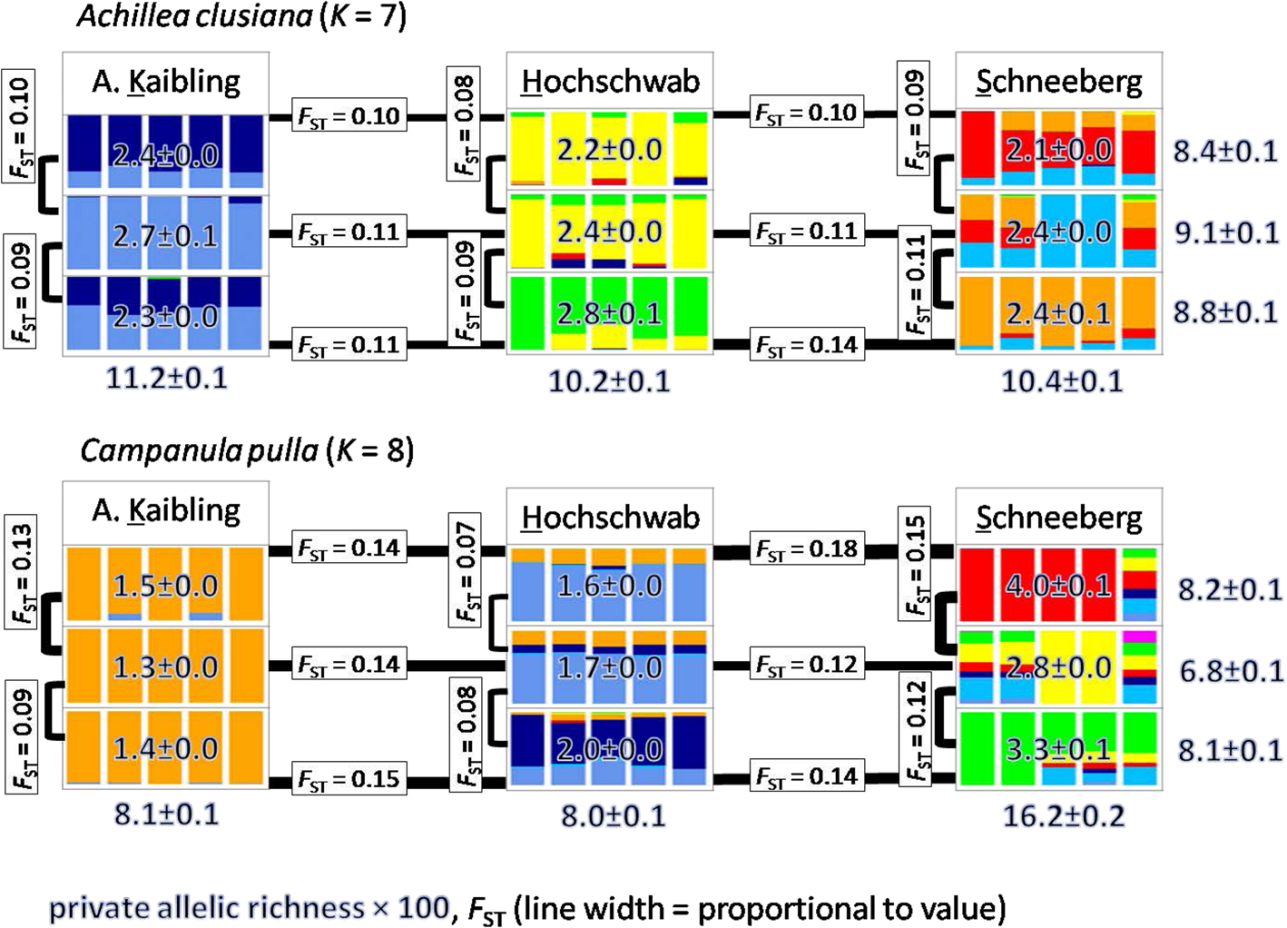
Graphical summary of STRUCTURE groups, observed private allelic richness and fixation index (*F*_ST;_ details in Fig. S7) of nine populations of *Achillea clusiana* (upper panel) and *Campanula pulla* (lower panel) in the Northeastern Calcareous Alps. Ancestry proportions are displayed for the best k per species. Mountains are displayed as they occur geographically from West to East. For each species, values for private allelic richness are given per mountain and elevational level (on top of the barplots for the elevational levels), per mountain (treating the upper, middle and lower elevational levels within mountains as only one population; below barplots for mountains), and per elevational level (treating the corresponding elevational levels in each of the three mountains as only one population; to the right of the barplots for elevational levels). Details see text

The following population genetics statistics to study diversity between and within populations of each species were computed with *Stacks*: observed and expected heterozygosities (*H*_I_, *H*_S_), nucleotide diversity (π), and inbreeding coefficient (*F*_IS_; Fig. 2 and Table S5).

Inbreeding leads to increased homozygosity and can be estimated using the inbreeding coefficient *F*_IS_, which is expected to be negatively correlated with heterozygosity. The inbreeding coefficient averaged over all populations was close to zero in *A. clusiana* and slightly negative in *C. pulla*, suggesting both species to be outcrossing. Mean *H*_I_ and *H*_S_ were slightly lower in *A. clusiana* than in *C. pulla*. Heterozygosity levels among *A. clusiana* subpopulations were relatively balanced (mean observed heterozygosity ranged from 0.0014 in the upper population on Hochschwab to 0.0016 in the middle population on the same mountain). In *C. pulla*, lowest *H*_I_ (0.0013) was observed in the medium population of Admonter Kaibling and highest *H*_I_ (0.0019) in the upper population from Schneeberg. Mean expected heterozygosity ranged from 0.0011 in the upper Admonter Kaibling population to 0.0016 in the lower Hochschwab population. *Campanula pulla* populations from Admonter Kaibling had lower than average *H*_I_ and *H*_S_ values. *H*_I_ was higher than *H*_S_ in all populations of both species, with the difference being most pronounced in the upper population of *C. pulla* at Schneeberg.

In *A. clusiana*, π was rather similar across the three mountains. In *C. pulla*, π was lowest on Admonter Kaibling and highest on Hochschwab. There was no clear elevational pattern in heterozygosity levels nor π in neither species.

Higher divergence within *C. pulla* than within *A. clusiana* was observed, reflected in higher per-site *F*_ST_ values (Fig. S6) and consistent with higher estimates obtained for *H*_I_, *H*_S_, and π. *F*_ST_ for each population pair of each species was estimated and visualized as heatmap to further investigate differentiation and dynamics of populations from the different elevations within each mountain and between mountains (Fig. S7). While differentiation between populations within each of the three mountains was at a similar level in *A. clusiana*, *F*_ST_ values were lowest among Hochschwab and highest among Schneeberg populations in *C. pulla.* Schneeberg populations, especially the lower population in *A. clusiana* and upper (but also lower) population in *C. pulla*, were the most diverged and thus showed the highest pairwise *F*_ST_ values with all other populations.

To account for disparity in the stacks coverage per sample (Table S4), private allelic richness was estimated using a rarefaction approach as implemented in *hp-rare* [56]. Private allelic richness was on average 0.0242 ± 0.0007 in populations of *A. clusiana*, and 0.0218 ± 0.0032 in populations of *C. pulla*, respectively. In *A. clusiana*, private allelic richness was in a similar range on all three mountains, whereas in *C. pulla*, it was approximately twice as high on Schneeberg as on the other two mountains (Fig. 2).

### Genetic structure and differentiation

**Fig. 3:**
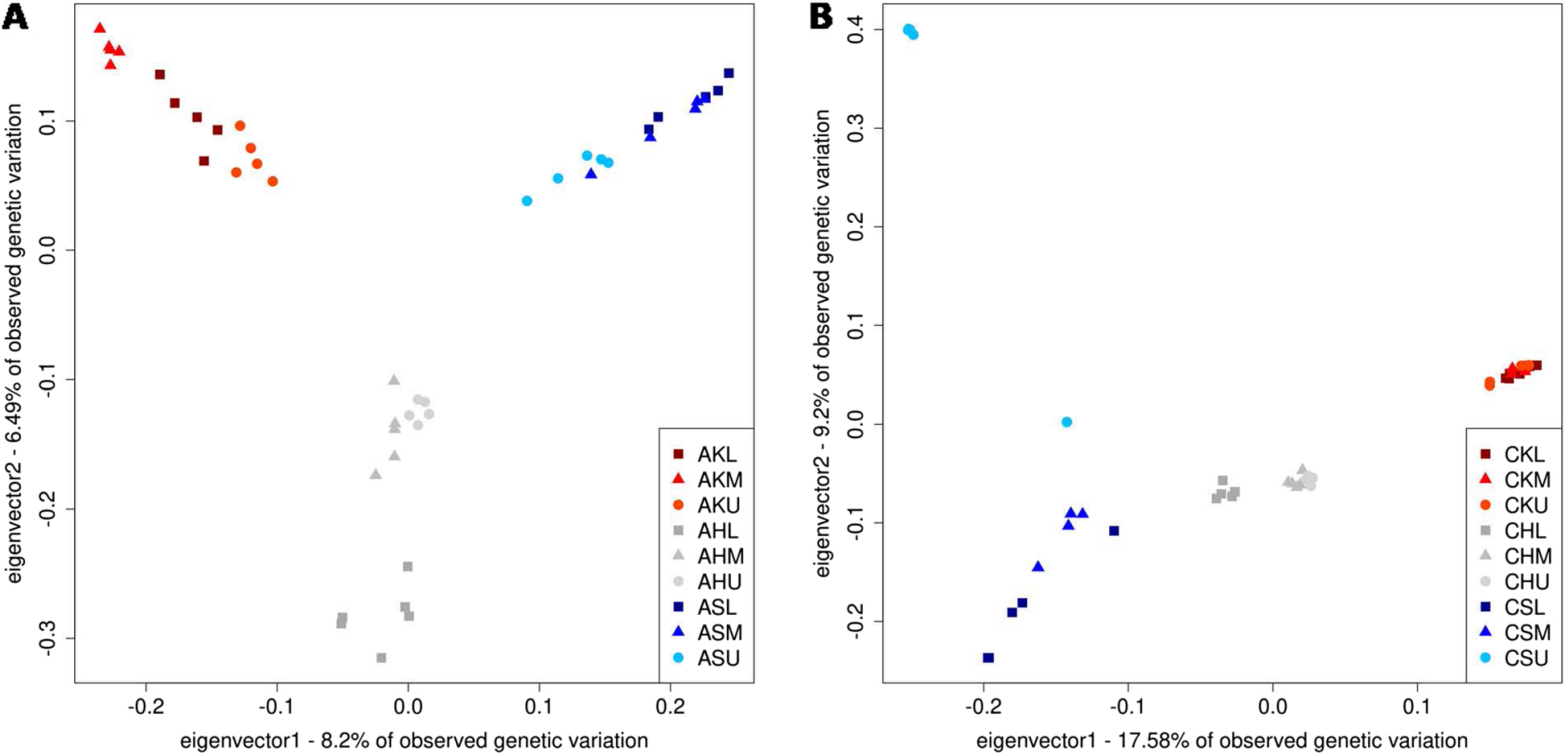
The first two components of a principal component analysis (PCA) based on allele frequencies observed for quality filtered variants obtained with *Stacks* for **A**) *Achillea clusiana* and **B**) *Campanula pulla*. Population names consist of species (A for *A. clusiana*, C for *C. pulla*), mountain (K for Admonter Kaibling, H for Hochschwab and S for Schneeberg) and elevation (lower L, medium M and upper U)

We performed a principal component analysis (PCA) to identify structure in our data based on the variation detected with *Stacks* for each species separately. Plotting the first two principal components (PC1 and PC2) showed a separation of populations from the three mountains in both species (Fig. 3).

In *A. clusiana*, PC1 and PC2 clearly distinguished the individuals sampled from the three mountains, with an east-to-west gradient (Admonter Kaibling – Hochschwab – Schneeberg) on PC1. Consistent with *F*_ST_ results (Fig. S7), the upper populations of the three mountains were closer to each other than the middle or lower ones. On Hochschwab, the cluster of *A. clusiana* individuals sampled at lower elevations was most distinct, whereas on Admonter Kaibling, the medium elevation population was distinct from the upper and lower populations (but still closer to the lower than to the upper population). In contrast to *A. clusiana,* we observed a stronger pattern of IBD rather than a clear differentiation by elevation in *C. pulla* (Fig. 3). A West–East cline was observed that is also reflected in highest *F*_ST_ between the westernmost mountain Admonter Kaibling and the easternmost mountain Schneeberg, with higher variability in the East (Fig. 2 and Fig. S7). PC1 differentiates the data by mountain and PC2 by elevation for Schneeberg populations (Fig. 3). Highest variation was observed within samples from Schneeberg, especially for individuals of the upper population. Consistent with the observations made for *A. clusiana* individuals along PC2, samples from Hochschwab were separated on PC1 with overlapping clusters of upper and medium populations suggesting gene flow between these two elevational levels, and a well separated cluster for samples from lower populations.

We used the identified quality filtered variants to generate a phylogenetic tree for each species using a maximum likelihood approach as implemented in *RAxML* [57]. Samples of both species formed monophyletic subtrees with high bootstrap support for each mountain and were generally grouped by elevation (Fig. S8).

### Admixture analysis

We used *STRUCTURE* [58, 59] to infer the presence of distinct populations and to identify potential migrants or admixed individuals in our dataset by testing three to nine *k* ancestral populations, with five replicates each. The resulting likelihoods per *k* were used as input to determine the best *k* according to the Evanno method [60] as implemented in *CLUMPAK* [61]. The best *k* was seven for *A. clusiana* and eight for *C. pulla*, respectively. The populations could be clearly distinguished by mountain and had different amounts of admixture (Fig. S9 and Fig. S10).

In *A. clusiana*, the admixture profile of the lower Hochschwab population was clearly different from the other two elevations (Fig. S9). On Admonter Kaibling we observed two co-dominant ancestries that were shared by the upper and lower populations, while in the medium-elevation population only one of these ancestries dominated clearly (Fig. S9). On Schneeberg, populations from each elevation showed distinct profiles with increasing *k*. The lower Hochschwab population had the most private alleles (Table S5). Intriguingly, we observed shared ancestry in *A. clusiana* upper populations of all three mountains, consistent with and further supporting PCA and *F*_ST_ results. This pattern, however, gets less pronounced with higher values of *k*.

The stronger pattern of IBD rather than separation by elevation that was observed before for *C. pulla* samples is also reflected in the admixture analysis. The ancestry profile of Hochschwab was a mixture of the ancestries dominating in Admonter Kaibling and of those that were weakly pronounced in Schneeberg individuals, which fits to its geographic location between them (Fig. S10). Interestingly, there were many variants with intermediate allele frequency in Hochschwab populations (Fig. S11). Consistent with results from the PCA the Admonter Kaibling admixture profile was homogeneous even between elevations and distinct from Hochschwab and Schneeberg based on the major ancestry proportion (Fig. 3 and Fig. S10). Within Hochschwab the low-elevation population was differentiated and within Schneeberg each elevation could be distinguished with increasing *k*, which also fits to the increased number of private alleles and more variants with intermediate frequency (Fig. 2, Fig. S10, Fig. S11 and Table S5).

### Environmental association analysis (EAA) of genomic regions under selection

**Fig. 4:**
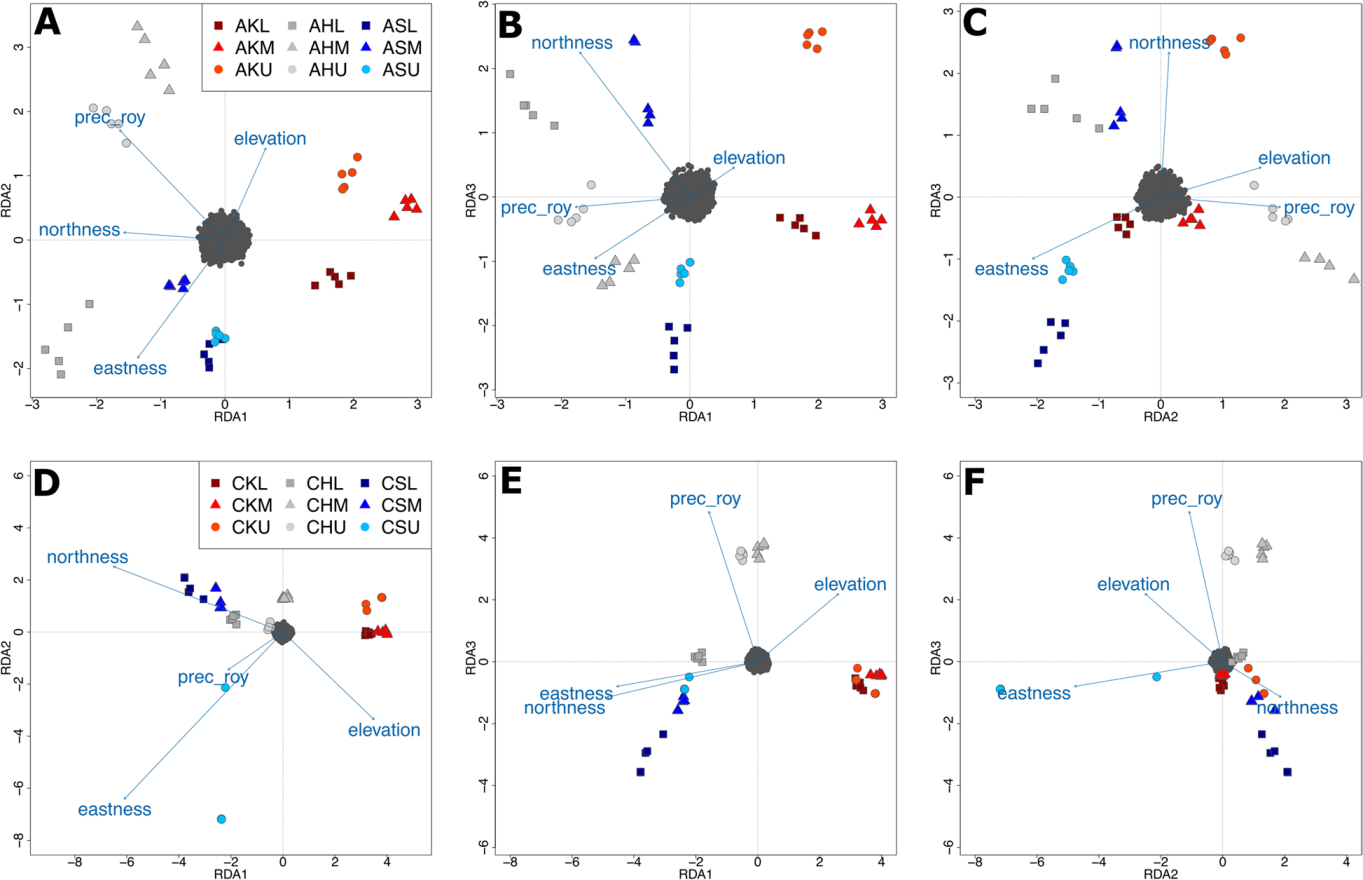
Triplots of the first three significant axis of the Redundancy Analysis (RDA) show variants (dark grey points), individuals (colored symbols) and environmental variables (arrows; eastness, elevation, northness and precipitation outside of the vegetation period (September–May; prec_roy)) in nine populations of *Achillea clusiana* (**A**–**C**) and *Campanula pulla* (**D**–**F**) in the Northeastern Calcareous Alps. Population names consist of species (A for *A. clusiana*, C for *C. pulla*), mountain (K for Admonter Kaibling, H for Hochschwab and S for Schneeberg) and elevation (lower L, medium M and upper U)

Redundancy Analysis (RDA) was employed to detect loci under selection and multilocus adaptation [62]. In *A. clusiana*, 95 single nucleotide variants (SNVs) under selection were significantly correlated with an environmental factor: 31 with September–May precipitation, 29 with northness, 23 with eastness and 12 with elevation (upper panel in Fig. 4 and Table S6). Seven of the twelve SNVs correlated with elevation were private to the upper population of Admonter Kaibling. In *C. pulla*, the number of SNVs under selection was much higher (370), the vast majority of which (209) were significantly correlated with eastness (82 of which were private to the upper population of Schneeberg and 1 to middle Hochschwab population), followed by September–May precipitation (150), northness (9) and elevation (2; lower panel in Fig. 4 and Table S6).

Triplots of the first three significant axes (RDA2 and RDA3 of *A. clusiana* had *p <* 0.002, all others had *p <* 0.001) of the RDA show SNVs (dark grey points), individuals (colored symbols) and environmental variables (arrows; Fig. S12). In *A. clusiana*, individuals from the middle and upper Hochschwab populations were positively and the lower population negatively related with September–May precipitation, whereas individuals from all other populations were more strongly related with exposition (i.e., eastness or northness; Table 2 and upper panel in Fig. S12). More specifically, Admonter Kaibling populations and Schneeberg lower and upper populations were characterized by southness (i.e., negative northness), the middle Schneeberg population by northness and the lower Hochschwab population by eastness. The pattern was almost identical in *C. pulla*, with the exception of the lower Schneeberg population (positively related with northness, negatively related with elevation) and the upper Schneeberg population (positively related with eastness; Table 2 and lower panel in Fig. S12). The Admonter Kaibling populations were additionally characterized by westness, and the lower Hochschwab population by northness, respectively.

**Table 2:** Population-level results of genome-environment association analysis in the alpine species *Achillea clusiana* and *Campanula pulla*

	*A. clusiana*		*C. pulla*	
Population	Axis with highest loading	Environmental factor	Axis with highest loading	Environmental factor
KL	1	**-N**	1	-E,** -N**
KM	1	**-N**	1	**-N**, -E
KU	1	**-N**	1	**-N**, -E
HL	1	** + E**, -P	1	** + E**, + N
HM	2	** + P**	3	** + P**
HU	2	** + P**	3	** + P**
SL^a^	3	-N	1	+ N, -ELE
SM	3	** + N**	1	** + N**, -ELE
SU	3	-N	2	+ E

Analysis based on Redundancy Analysis (RDA). Given are axis with the highest loading and environmental factors associated with a given population. Environmental factors coinciding in the two species printed in bold

*ELE* Elevation, *E* Eastness, *N* Northness, *P* Sep–May precipitation, Populations: *K* Admonter Kaibling, *H* Hochschwab, *S* Schneeberg, *L* lower, *M* Middle, *U* Upper elevation

± indicate positive/negative relationship with environmental factor

^a^note that the lower Schneeberg populations of *A. clusiana* and *C. pulla* differ in elevation by 136 m

## Discussion

In this study we assessed the suitability of two alpine species, *Achillea clusiana* and *Campanula pulla*, to serve as models for genetic monitoring of climate change adaptation along an elevational gradient. Furthermore, we identified loci under selection and correlated them with environmental factors relevant in a climate change context.

### Genetic diversity and differentiation along the elevation gradient

Our results clearly show, on the one hand, that the three mountains studied harbored a distinct gene pool for each of the two model species and, on the other hand, that the populations were mostly differentiated within a mountain range along the elevation gradient. Populations from Schneeberg and Admonter Kaibling showed the same pattern of elevational differentiation in *A. clusiana* and in *C. pulla*, namely that the upper population was sister to a group formed by the middle and lower populations in the phylogenetic analysis (with 96–100% bootstrap support in Fig. S8). Populations from Hochschwab deviated from this pattern. In *A. clusiana*, the Hochschwab populations formed a clearly delineated group, but unlike in the other two mountains, the lower population was sister to a group formed by the middle and upper populations (but with little support). In *C. pulla* we found an even more different pattern. The Hochschwab populations did not form a separate group at all but were laddered between populations from Schneeberg and Admonter Kaibling. Successful genetic monitoring requires that both the three mountains and the three populations along the elevation gradient within a mountain have an independent genetic profile. The former could be clearly affirmed, even if the Hochschwab populations of *C. pulla* had a higher level of admixture. The second was only partially affirmed, since not all elevation levels showed an equally independent profile. Our expectation that the upper populations would have higher genetic diversity due to a more extensive habitat was not confirmed, as populations from all elevation levels had similar diversity. We assume that the fragmented nature of the snowbed habitat of the two species is responsible for this pattern. Similarly, other studies found that within-population genetic diversity was mostly not related to elevation (and population size) in alpine plants [63, 64]. Generally, alpine species show higher genetic differentiation among populations and lower genetic diversity than species from lower elevations [65].

### Past and present gene flow

To interpret current diversity and differentiation among populations, it is also necessary to consider the history of the populations. All three of the studied mountain ranges were located east of the completely glaciated area of the Alps during the last ice age and are considered refugial areas for alpine plants [40]. This view is supported by our results: the fine-resolution GBS method showed that each of the three mountain ranges probably provided a separate refugium for alpine plants. For *A. clusiana*, all three mountains appear to have played a similarly important role as refugia, as the populations from each mountain had a similarly high value of private allelic richness [42]. For *C. pulla*, Schneeberg even appears to have provided two separate refugia, one whose gene pool was represented by all but one individual from the upper population (CSU141–CSU144), and a second whose gene pool was represented by the remaining Schneeberg individuals. For *C. pulla*, Schneeberg thus seems to have played a more important role than the other two mountains, which is reflected in the twice as high private allelic richness of Schneeberg populations.

It is assumed that the alpine vegetation zone of the Alps shifted downwards by about 1500 m following the climatic snowline in the north-eastern Alps during the last ice age [41] which is substantially below the present lower populations of our model species. Thus, mutations which had accumulated in refugial populations during the glacial period may have been lost during the postglacial upward migration and it is expected that today's lower populations still have a greater proportion of such private mutations than today's upper populations. To some extent, we observed this trend in our analyses. In *A. clusiana*, the lower and/or middle populations have the highest private allelic richness and the upper the lowest. At Admonter Kaibling, where the middle population has the highest private allelic richness, the situation is special in that the lower population is at the bottom of a steep ravine. Due to frequent and intensive disturbance by debris flows, the population appears to have been repeatedly destroyed and re-established. In *C. pulla*, the expectation of highest private allelic richness in the lower population (and lowest private allelic richness in the upper population) was confirmed only at Hochschwab. We interpret the pattern at Schneeberg as an indication of two separate refugia, each with a comparatively high private allelic richness, suggesting large refugial populations, possibly favored by the location on the edge of the Alps. At Admonter Kaibling, on the other hand, all three populations are relatively uniform, also in terms of private allelic richness. However, although they had the smallest population sizes (pers. obs. M. Winkler & K. Tremetsberger), the lower populations harbored a substantial proportion of the private alleles (about a third of the total private allelic richness in both species; Fig. 2), which may be irretrievably lost if the lower populations disappear as a consequence of climate change and the upwards shift of the tree line [66] leading to the loss of any adaptive potential to climate change they may convey.

Interestingly, in *A. clusiana* PCA and pairwise *F*_ST_ estimates showed that the upper elevation populations were genetically closest to each other (Fig. 2, Fig. 3 and Fig. S7). *Achillea clusiana* populations from lower elevations thus seem to have smaller *N*_e_ and stronger drift. Admonter Kaibling is the only exception where the medium population is more distant to the upper than the lower elevation population. This population grows above the subalpine vegetation belt in a small ravine in a steep rock face and consists of a small number of individuals. The assumption of smaller *N*_e_ and greater drift at lower and medium elevations is plausible considering postglacial reforestation and the habitats of the lower populations in the subalpine *Pinus mugo* krummholz zone (in shady dolines on Hochschwab and Schneeberg, at the bottom of a steep ravine with long snow cover on Admonter Kaibling). Another explanation for our observation that populations from upper elevations are closer to each other would be gene flow directly between the summit regions of the mountains. Although neither study species is specifically adapted to wind dispersal, it is conceivable that infrequent diaspore dispersal between mountain tops could occur with blustery winds. Furthermore, pollinator insects already loaded with pollen may be displaced by stormy winds or diaspores may be transported by birds.

Because of the two presumed glacial refugia at Schneeberg, long-term connections between *C. pulla* populations were more complex. The upper Schneeberg population (except for individual CSU145; see below) was the most differentiated from all other populations. The population is located on the southern flank of Schneeberg, which is not oriented towards the other two mountains. If we disregard the upper Schneeberg population, we can observe the same pattern of decreasing pairwise *F*_ST_ between populations of the same elevation from lower to upper elevation as in *A. clusiana*. We interpret this as another result of smaller population sizes and stronger drift in the lower populations growing in a shaded doline in the subalpine krummholz zone on Hochschwab, in a steep rock face on Schneeberg, and in the "Eisloch" at the bottom of a steep ravine on Admonter Kaibling. At Hochschwab, the stronger differentiation between the lower population (located on the north-eastern part of the Hochschwab massif) and the middle and upper populations (in the center of the Hochschwab massif) was evident in both *A. clusiana* and *C. pulla* (Fig. 3). The greater horizontal distance between these subpopulations on Hochschwab may be responsible for this observation, in addition to the difference in elevation.

One of the 45 studied individuals of *C. pulla* (CSU145) may be a descendant of a recent migrant that dispersed from the middle population at Schneeberg to the upper population a few generations ago and interbred with the local individuals there. The presumed dispersal was from near the middle Schneeberg population, i.e., from about 1860 m a.s.l. about 800 m in an SSE direction to an SE-exposed scree slope immediately below the ridge and not far from a dirt road (2030 m a.s.l.). Another way to look at our result is that individuals CSU141–CSU144 may have recently immigrated from an unsampled, genetically divergent population. If they arrived through a clustered migration event (e.g., the entire capsule was dispersed), this would explain their striking genetic proximity. In any case, a mixture of two divergent gene pools was observed in the upper Schneeberg population, apparently the result of a recent migration event. No presumed descendants of recent migrants were sampled in *A. clusiana*. The results show that the GBS method is very sensitive to detect recent migration events and that such events occur between populations of a mountain. However, to estimate the frequency of such events, it would be necessary to genotype many more individuals.

### Genome-environment associations

The populations of the two species showed an almost identical pattern of correlation of SNVs under selection with environmental variables, with September–May precipitation being most important for Admonter Kaibling middle and upper populations, and exposition (eastness or northness) for all other populations suggesting similar selective pressures on the two snowbed species. The only exception to this common pattern was the upper Schneeberg population, with *A. clusiana* showing a negative relationship with northness, and *C. pulla* a positive relationship with eastness. The lower Schneeberg populations, which also showed a divergent pattern, cannot be meaningfully compared as the two species were sampled in different localities more than 100 altitudinal meters apart from each other.

Both September–May precipitation and exposition influence snow depth and the timing of snow-melt. At high elevations, precipitation outside the vegetation period predominantly falls in the form of snow, with snow accumulating in depressions formed in the Karst landscape of the calcareous study sites. The exposition on the other hand determines exposure to wind, and has also thermic consequences. In the Alps, (north)westerly winds prevail, with snow accumulating at the lee side [67]. Furthermore, southern and eastern mountain slopes are warmer, harbor more species and have more colonization events than northern and western slopes [68]. In contrast to our expectations, temperature (represented by elevation in the RDA analysis, which was highly correlated with temperature variables; Table S7) seems to play only a minor role in local adaptation. This suggests that the consequences of climate change will predominantly manifest via changes in precipitation and, thus, duration of the snow cover in the snowbeds and indirectly via shrub encroachment (e.g., [14]) accompanied by increasing shading of snowbeds at the lower range margins. For instance, in the central Alps, high-elevation vegetation composition has been changing towards species adapted to drier conditions. This compositional change has accelerated in the past decades [69].

Species distribution models project considerable range losses for both our study species by the end of the century, especially under more severe climate change [70]. However, while these models account for demography and seed dispersal, they assume a single optimal climatic niche for the entire species without local adaptation. Accounting for eco-evolutionary dynamics, Cotto et al*.* (2017; [71]) predict that long-living adults persist in an unsuitable climate preventing rapid turnover and producing increasingly maladapted offspring, leading to a decrease in population size before a range size reduction. Furthermore, due to established cold-adapted individuals blocking colonization from lower elevations, the frequency of warm-adapted genotypes is predicted to decrease with increasing warming [72]. However, Gonzalo-Turpin and Hazard (2009; [73]) have shown for *Festuca eskia* in the Pyrenees that local adaptation of alpine plants occurred in response to “harsh” conditions at higher elevation rather than to “mild” conditions at lower elevation and that such adaptation exists despite of gene flow that may occur through pollen or seed. Similarly, in *Arabis alpina* it is unclear whether gene flow from low-elevation populations, e.g. in the form of pollination, will be able to rescue increasingly maladapted high-elevation populations [64]. Therefore, the possibility of evolutionary rescue in the face of climate change of high- and low-elevation populations of *A. clusiana*, *C. pulla* and other alpine plants remains an important topic for further investigation.

## Conclusions

The aim of the present study was to evaluate the suitability of *A. clusiana* and *C. pulla* as model species and of GBS as a method for genetic monitoring. The biological characteristics of the species, such as a diploid chromosome set and low levels of inbreeding, indicate that the species are suitable. For genetic monitoring of climate change adaptation, it is necessary to detect rare recent dispersal events. The GBS method seems to provide sufficient resolution for this in most cases. The population histories of the two species differ to some extent, but this is not a hindrance to studying the question of adaptation to climate change. However, because GBS reveals only a small portion of the genome and predominantly neutral variation, other genetic methods are needed to trace the spread of adaptive variants with climatic change. One would have to assemble the genomes of the two species. Meanwhile, it would also be (financially) feasible to re-sequence the entire genomes of hundreds of individuals.

The results obtained herein suggest that a mere space-for-time substitution approach helps drawing some conclusions but may not be sufficient to assess the consequences of climate change on the evolutionary potential of alpine plant species as the genetic signature is not only formed by present environmental and demographic factors but also by population history [74]. Follow-up studies on larger sample sizes and time series will be necessary to functionally characterize and validate these newly identified loci putatively involved in adaptive processes.

## Methods

### Study species

*Achillea clusiana* Tausch (Asteraceae), a species endemic to the Northeastern Calcareous Alps, belongs to *Achillea* sect. *Anthemideae* and is closely related to the more widespread *A. atrata*. Both species are reported to have a diploid genome (2*n* = 18; [52, 53]). *Achillea clusiana* is a perennial rosette plant with clonal growth and is highly aromatic [75]. The species is obligatorily outcrossing [76] and pollinated by insects, achenes are approximately 2 mm long without pappus. A median of 41% of the individuals are flowering, with a median seed yield of 91 seeds per flowering individual [70]. *Achillea clusiana* occurs in the subalpine to alpine zone on moist calcareous screes and snowbeds [75].

*Campanula pulla* L. (Campanulaceae) is an endemic of the Northeastern Alps, where it occurs in the same habitat types as *A. clusiana* [75]. *Campanula pulla* is perennial with limited clonal growth. Approximately 56% of the individuals are fertile, each producing on average 173 small seeds with no apparent specialized dispersal mechanism [70].

### Climate data

CHELSA provides high-resolution datasets for temperature and precipitation on a ~ 1 km grid worldwide [47, 77, 78]. We extracted monthly means for precipitation and minimum, maximum and average temperature (Tmin, Tmax, Tmean) for each CHELSA cell containing our sampling locations from 1979 to 2013. For downscaling these estimates to the specific spot of a sampling locating, we used a statistical downscaling procedure called “delta method”, which has frequently been applied in studies of climate change effects (e.g., [79, 80]). To get a better representation of the climate surface to the study terrain we first refined the ~ 1 km resolution CHELSA climate data to 100 m resolution based on the dependency of precipitation and temperature on elevation by means of linear regressions in moving windows (for a detailed description of this step see [80]). The delta method consisted then of the following steps: (1) calculate long-term mean values (1979–2013) for precipitation and temperature for each month and each CHELSA cell; (2) calculate anomalies (deltas) as the differences between annual climatic values and the long term mean (1979–2013) using the ratio for precipitation and absolute differences for temperatures; (3) multiply (for precipitation) respectively add (for temperature) these anomalies to the high-resolution CHELSA climate data.

### DNA extraction and sequencing

Collected leaf samples were dried in silica gel. DNA was extracted from 15–30 mg dried leaf tissue using the DNeasy Plant Mini Kit extraction kit (Qiagen, Hilden, Germany) following the manufacturer’s protocol, but adding two sorbitol washing steps after leaf tissue disruption. DNA quality was checked on agarose gels. DNA concentrations were quantified with a DS-11 FX Fluorometer (DeNovix, Wilmington, Delaware, USA), using the dsDNA Broad Range Assay Kit (DeNovix) according to the manufacturer’s manual. DNA was dried and shipped for library preparation and sequencing to The Elshire Group Ltd., New Zealand. The sequencing data was generated following a modified version of the protocol by [48] that included the following changes: 100 ng of genomic DNA were used, 3.6 ng of total adapters were used, the genomic DNAs were restricted with *Ape*KI enzyme and the library was amplified with 18 PCR cycles. Combinatorial bar codes were used to generate the libraries, and 150 nucleotides long paired end reads were generated by Illumina sequencing.

### Preparing RAD-seq data for downstream analyses

Raw sequencing data were demultiplexed to obtain per-sample data files. As recommended by [48], *axe-demux* [81] was used with barcodes and sample IDs provided in the keyfile. In total, 486 million read pairs or 95.1% of the raw sequencing data contained valid barcodes and could thus be demultiplexed.

Barcoded adapters were removed from forward and reverse sequencing reads using the Perl script batch_trim.pl [82], in combination with barcodes and sample IDs provided in the keyfile. The last and first three bases of each read were removed with *cutadapt* (-u 3 -u -3 -U 3 -U -3; [83]) to ensure removal of all CWG overhangs of the cutting enzyme *Ape*KI.

*FastQC* [84] was used to identify overrepresented sequences. *BLAST* [85, 86] revealed high homology to putative contaminants or other unwanted sequences, like cpDNA, mtDNA or bacterial, viral or human DNA. Overrepresented sequences were extracted from the *FastQC* report and *bwa mem* [87] was used to map them back to the sequencing data. *SAMtools* [88, 89] was used to extract only those read pairs with zero matches for further analyses. This procedure was repeated until final quality checks with *FastQC* showed that no overrepresented sequences remained in the data.

For analysis with *Stacks* [54] it was necessary to further trim the reads to equal lengths of 90 nt. Trimming was performed with *Trimmomatic* [90] using the settings CROP:90 MINLEN:90.

### Variant-based analyses

*Stacks* can be used to analyze RAD-seq data either with or without a reference genome. In the case of a missing reference sequence, *Stacks* assembles reference loci de novo from RAD-seq data and then uses these loci to call variants from the sequencing data. Several population genetic analyses are implemented in *Stacks*, but can also be performed individually with other tools as *Stacks* is able to print different output formats. For analysis with *Stacks* a series of sub-programs has to be run for sequencing reads trimmed to equal length, which in our case were 90 nt (*ustacks*, *cstacks*, *sstacks*, *tsv2bam*, *gstacks* and *populations*; Table S3).

Different *Stacks* settings were tested for assembling and variant calling performance per species. Values one to eight were tested for each of the following parameters, while default settings for all other parameters were used: *ustacks* -m (default 3), -M (default 2) and -N (default M + 2) and *cstacks* -n (default 1; suggested: set to *ustacks* -M). Performance of these parameter combinations was tested for each *population* parameter-pair -r from 0.4 to 1.0 in 0.2 increments and -p from one to nine. In the end, slightly modified *Stacks* assembly and variant analysis settings according to the recommendation from [91] were applied (*ustacks* -m 3, -M 2 and -N 2 and *cstacks* -n 2 and *populations* -r 0.8, -p 9 and -min-maf 0.05), as described in detail below.

Except for -N 2 (default: M + 2), default settings were used to run *ustacks*; -N determined the maximum distance allowed to align secondary reads to primary stacks and was lowered compared to the default in order to stronger punish secondary reads, that is reads that did not form putative *Stacks* loci; -M 2 determined the maximum distance allowed between stacks and -m 3 determined the minimum depth of coverage required to create a stack.

Except for -n 2 default settings were used to run *cstacks*; -n determined the number of mismatches allowed between sample loci when building the catalog.*gstacks* was used to incorporate the paired-end reads, as fetched by *tsv2bam*, and to assemble paired-end reads into contigs (non-overlapping read pairs are connected via an N-decamer by *Stacks*), to merge contigs with the respective single-end locus, to align reads from individual samples to the locus, to identify SNVs and to genotype each individual at each variant.

Output files from *gstacks* were used as input for *populations* to obtain estimates of heterozygosity, diversity and fixation indices and the variant and allele frequency files that were used for phylogenetic and admixture analysis and PCA and EAA. Except for -p 9, -r 0.8 and -min-maf 0.05 default settings were used; -p determined the minimum number of populations a locus must be present in to process a locus, -r determined the minimum percentage of individuals in a population required to process a locus for that population and -min-maf specified the minimum minor allele frequency required to process a SNV.

The patterns observed in pairwise *F*_ST_ were robust and consistent between results obtained using different *Stacks* parameters and independent of the *populations* parameter -write-random-SNV, which can be useful to avoid effect of linkage disequilibrium (LD) on downstream analyses that are sensible to this phenomenon, like *F*_ST_ calculations, by randomly selecting only one variant site per locus for the estimations.

The resulting quality filtered variants were used to perform phylogenetic and admixture analysis and PCA and EAA as described below.

### Ploidy estimation

*nQuire* [50] was used for ploidy estimation of each analyzed sample using the sorted and collated matches to the *Stacks* locus catalog of the respective species. The denoise option was used to counteract noise resulting from mismappings, which reduced the number of considered sites by 65% in *A. clusiana* and by 61% in *C. pulla*. Visual inspection of the ploidy levels clearly supported the diploid status of all tested individuals independent of denoising, i.e. base frequency peaks in results from the *histo* command were always at 0.5 and the diploid model always had best fit between ideal and empirical histograms as characterized by lowest sums of squared residuals of empirical vs. ideal histograms, positive slope with low standard error and highest correlation coefficients of the regression of y-values in results from the *histotest* command (averages for the denoised runs were r^2^(2N) = 0.12, r^2^(3N) = 0.01, r^2^(4N) = 0.02 in *A. clusiana* and r^2^(2N) = 0.14, r^2^(3N) = 0.02, r^2^(4N) = 0.02 in *C. pulla*, respectively).

### Genome size measurements

To measure the genome size of the two model species by flow cytometry, we used the CyStain PI Absolute P kit (Sysmex, Görlitz, Germany) according to the manufacturer's instructions with leaf tissue of the sample and parsley (*Petroselinum crispum* (Mill.) Fuss, 1C = 2.23 pg; [92]) or pea (*Pisum sativum* L. ‘Kleine Rheinländerin’, 1C = 4.42 pg; [93]) as internal standard. At least 5,000 measurements were gathered in a CyFlow Space (532 nm diode laser; Sysmex) to estimate nuclear DNA content. Two individuals of *A. clusiana* and three individuals of *C. pulla* (all from Hochschwab) were measured. One individual of each species was measured twice, one time with parsley as standard, one time with pea as standard. The remaining individuals were measured once, with pea as standard in the case of *A. clusiana* and with parsley as standard in the case of *C. pulla*.

### Principal component analysis (PCA)

PCA is a dimension-reducing method that summarizes the variation observed in the data as linear combinations of multivariate observations. For each component, PCA assigns weights to the traits (variants) that contribute most to the variation observed in the data. Samples can then be plotted along the resulting orthogonal axes to identify clusters that represent biological groups. PCA has become a popular tool for exploring multilocus population genetics data to study population structure and identify migrants and hybrids or polyploids. The quality filtered variants called with *Stacks* were printed with *populations -vcf* in.vcf format and further used to perform PCA with *smartPCA* [94] to detect cluster of samples. The genotypefile, SNVfile, indfile and parfile needed to run *smartPCA* were generated with *bcftools query* [88] and custom bash and python scripts. Results were visualized using *R*.

### Phylogenetic analysis

The quality filtered variants (see above) were used as input to generate maximum likelihood trees with *RAxML* to study phylogenetic relationships in the data. *bcftools query* was used to extract genotypes and then bash and python were used to generate pseudohaploid multifasta alignments by randomly sampling allele 0 or allele 1 in case of heterozygous sites, and to convert the file to a format readable by *RAxML*. The GTRCAT model was used and the resulting bootstrap values of 1,000 bootstrap replicates annotated to the respective maximum likelihood trees. The resulting trees with bootstrap values were midpoint-rooted and visualized in *figtree*.

### Admixture analysis

A well-established approach to effectively study population structure in biological multilocus genotype data is implemented in *STRUCTURE* [58, 59]. In this model-based method a number of *k* ancestral populations is assumed where *k* in general is unknown. Individuals get probabilistically assigned to each *k* based on sets of observed allele frequencies at each locus. Depending on the level of admixture, individuals thus are assigned to one population in case of no mixing, or jointly to two or more populations if their genotypes indicate that they are admixed. Within populations, Hardy–Weinberg equilibrium is assumed and weak linkage between alleles is accounted for. *STRUCTURE* is implemented in a Bayesian framework and for each tested *k* a likelihood is obtained that is then compared to other settings of *k* to get an estimate of the* k* that best represents the true number of ancestral populations represented by the analyzed individuals.

The *populations -structure* command was used to get the *Stacks* variants in a format readable by *STRUCTURE*. We used an admixture model, accounted for linkage and tested *k* three to nine for each species. We ran five independent Markov chain Monte Carlo (MCMC) operations (200,000 iterations after a burn-in of 100,000) for each *k*. The best *k* for each species was evaluated using the Evanno method [60] as implemented in *CLUMPAK* [61, 95].

### Environmental association analysis (EAA)

We conducted a genome-environment association analysis using Redundancy Analysis (RDA), a multivariate method to detect loci under selection and multilocus adaptation [62]. In the quality-filtered variant dataset of *A. clusiana* and *C. pulla* a total of 3.5% and 2.3%, respectively, were missing values. As RDA does not allow any missing data the analysis was restricted to only a proportion of the total variation observed (29% and 44% in *A. clusiana* and *C. pulla*, respectively). Environmental variables we considered were elevation, eastness, northness, and the following downscaled climate variables: annual precipitation, precipitation in the vegetation period (Jun–Aug) and in the rest of the year (Sep–May; prec_roy), mean annual temperature, and minimum, maximum and mean monthly temperatures of June to September. To minimize collinearity we reduced the set of variables entered in the analysis to elevation, eastness, northness and precipitation outside of the vegetation period (Sep–May; prec_roy; Table S1). Elevation was highly correlated with all temperature variables (|r|> 0.94; Table S7) and can thus be considered a surrogate for temperature. September–May precipitation was chosen over annual precipitation and precipitation in the vegetation period as it was least correlated with elevation (Table S7). Eastness was not correlated (|r|< 0.11), and northness only weakly correlated (|r|< 0.47) with the other environmental variables. Correlation coefficients were estimated using the R function *cor.test*.

## Supplementary Information

**Additional file 1:** **Table S1.** Environmental variables characterizing populations of A) *Achillea clusiana* and B) *Campanula pulla* analyzed in the study. MAT… mean annual temperature, T… temperature.

**Additional file 2:** **Table S2.** Minimum, maximum and average number of reads in million left per sample after the different filtering steps per species and in total. Filter1 is demultiplexing, filter2 is removing adapter sequences, filter3 is removing *Ape*KI restriction enzyme overhangs (CWG), filter4 is removing reads with overrepresented sequences as identified with *FastQC* and filter5 is trimming reads to 90 nt length such that they can be used with *Stacks*.

**Additional file 3:** **Table S3.** Per sample and species summary statistics of the sequencing data and after each of the five filtering steps. Columns show basecount, readcount, % of bases left, % of reads left. Filter1 is demultiplexing, filter2 is adapter removal, filter3 is trimming of *Ape*KI cutting enzyme overhangs (CWG), filter4 is removal of overrepresented sequences and filter5 is trimming to 90 nt.

**Additional file 4:** **Table S4.** Summary statistics of the *Stacks* analysis per sample and species. Columns show the number of input reads used to run *Stacks* per sample and in total per species, the number of stacks generated by *Stacks* (*ustacks*) and the stacks coverage per sample and on average per species and the per sample min., average, max. and SD per species.

**Additional file 5:** **Table S5.** Summary statistics of the *Stacks* analysis per population. Average values per species and of populations from each elevation and mountain per species and in total are given. The number of polymorphic sites and private alleles are listed as well as several population genetics statistics: observed and expected heterozygosity (*H*_I_ and *H*_S_), nucleotide diversity (π) and inbreeding coefficient (*F*_IS_). The other six columns show the number of polymorphic loci and sites and private alleles after each of the two variant filtering steps. First it was filtered for genotype quality and read depth. The second filter included removal of duplicated *Stacks* loci.

**Additional file 6:** **Table S6.** Summary statistics of all variants significantly correlated with an environmental variable for A) *Achillea clusiana* and B) *Campanula pulla*. Analysis based on Redundancy Analysis with the environmental variables eastness, elevation, northness and precipitation outside of the vegetation period (May–Sep; prec_roy). Columns show significant axis, variant coordinate, variant loading, correlation with each predictor and correlation of predictor that most strongly correlates with variant. Variants that are private to a population are marked. Genotypes for all samples are listed. Sample and population names: A ... *A. clusiana*, C ... *C. pulla*; H ... Hochschwab, K ... Admonter Kaibling, S ... Schneeberg; L … population at lower elevational range limit, M … intermediate, U … upper elevational range limit.

**Additional file 7:** **Table S7.** Pearson correlation coefficients between environmental variables of nine populations of *Achillea clusiana* (upper diagonal) and *Campanula pulla* (lower diagonal) in the Northeastern Calcareous Alps. Climate variables were downscaled from CHELSA data 1979–2013. MAT… mean annual temperature, T… temperature.

**Additional file 8:** **Figure S1.** Percentage of raw reads left after the different filtering steps for samples of each A) *Achillea clusiana *and B) *Campanula pulla *population. Filter1-3 corresponds to 100% and is the data after demultiplexing, adapter removal and trimming of CWG overhangs from *Ape*KI digestion, filter4 is removal of overrepresented sequences and filter5 is trimming to 90 nt. For each species the per-sample average per filtering step is included as dashed line. Population names consist of species (A for *A. clusiana*, C for *C. pulla*), mountain (K for Admonter Kaibling, H for Hochschwab, and S for Schneeberg) and elevation (lower L, medium M, and upper U).

**Additional file 9:** **Figure S2.** Distribution of read lengths after applying filter1-3 (demultiplexing, adapter removal and trimming of CWG overhangs from *Ape*KI digestion) for A) *Achillea clusiana *and B) for *Campanula pulla*. Mean and median read lengths are indicated.

**Additional file 10:** **Figure S3**. Stack coverage of stacks per population of A) *Achillea clusiana *and B) *Campanula pulla*. Stacks were generated with the *ustacks *step of the *Stacks *pipeline. The per-species average coverage is indicated with a dashed red line. Population names consist of species (A for *A. clusiana*, C for *C. pulla*), mountain (K for Admonter Kaibling, H for Hochschwab, and S for Schneeberg) and elevation (lower L, medium M, and upper U).

**Additional file 11:** **Figure S4.** Distribution of observed stack read depths (DP; A) and C)) and observed genotype qualities (GQ; B) and D)) for the variants detected in *Achillea clusiana *in the upper panel and *Campanula pulla *in the lower panel, respectively. Thresholds for genotype quality and read depth filtering of the variants were set per species according to these plots: GQ 32 and DP 4–36 in case of *A. clusiana* and GQ 33 and DP 4–53 in case of *C. pulla*, respectively.

**Additional file 12:** **Figure S5.** Length distribution of final quality filtered *de novo *assembled polymorphic *Stacks* contigs for *Achillea clusiana *in A) and for *Campanula pulla *in B), respectively. Mean and median read lengths are indicated.

**Additional file 13:** **Figure S6.** Distribution of per-site *F*_ST_ values for each quality filtered variant for A) *Achillea clusiana *and B) *Campanula pulla*. Mean and median *F*_ST_ values are indicated.

**Additional file 14:** **Figure S7.** Pairwise *F*_ST_ values (below the diagonal) for all populations based on variants obtained with Stacks for A) Achillea clusiana and B) for Campanula pulla. Numbers of shared variants are shown in grey above the diagonal. Population names consist of species (A for A. clusiana, C for C. pulla), mountain (K for Admonter Kaibling, H for Hochschwab, and S for Schneeberg) and elevation (lower L, medium M, and upper U).

**Additional file 15:** **Figure S8.** Midpoint-rooted maximum likelihood tree based on quality filtered variants for A) *Achillea clusiana* and B) for *Campanula pulla*. Branches are supported by bootstrap values obtained from 1,000 replicates. Sample names consist of species (A for *A. clusiana*, C for *C. pulla*), mountain (K for Admonter Kaibling, H for Hochschwab, and S for Schneeberg), elevation (lower L, medium M, and upper U) and the individual sample ID.

**Additional file 16:** **Figure S9.** Admixture results obtained with *STRUCTURE* assuming *k* three to nine ancestral populations and using allele frequencies for variants obtained with *Stacks* for *Achillea clusiana*. Samples are sorted from left to right starting with the westernmost mountain Admonter Kaibling, over Hochschwab to the easternmost mountain Schneeberg and populations of each mountain are sorted by elevation in increasing order from left to right. Ancestry proportions for each sample are shown on the y-axis. Sample names consist of species (A for *A. clusiana*), mountain (K for Admonter Kaibling, H for Hochschwab, and S for Schneeberg), elevation (lower L, medium M, and upper U) and the individual sample ID.

**Additional file 17:** **Figure S10.** Admixture results obtained with *STRUCTURE* assuming *k* three to nine ancestral populations and using allele frequencies for variants obtained with *Stacks* for *Campanula pulla*. Samples are sorted from left to right starting with the westernmost mountain Admonter Kaibling, over Hochschwab to the easternmost mountain Schneeberg and populations of each mountain are sorted by elevation in increasing order from left to right. Ancestry proportions for each sample are shown on the y-axis. Sample names consist of species (C for *C. pulla*), mountain (K for Admonter Kaibling, H for Hochschwab, and S for Schneeberg), elevation (lower L, medium M, and upper U) and the individual sample ID.

**Additional file 18:** **Figure S11.** Allele frequencies for quality filtered variants for A) *Achillea clusiana* populations and B) *Campanula pulla* populations. Allele frequency range was split into ten bins of bin size ten with stepsize ten. Population names consist of species (A for *A. clusiana*, C for *C. pulla*), mountain (K for Admonter Kaibling, H for Hochschwab, and S for Schneeberg) and elevation (lower L, medium M, and upper U).

**Additional file 19:** **Figure S12.** Triplots of the first three significant axis of the Redundancy Analysis (RDA) show variants (grey open circles) and environmental variables (colored arrows; eastness, elevation, northness and precipitation outside of the vegetation period (September–May; prec_roy)) for *Achillea clusiana* (A–C) and for *Campanula pulla* (D–F), respectively. Variants are colored depending on which environmental variable they significantly correlate with.

## Data Availability

The demultiplexed GBS data generated for this project are deposited at the NCBI SRA archive (BioProject accession: PRJNA847734). The de novo assembled loci, called variants, keyfile and detailed code for the analyses including a list of used software and versions are available on figshare [96].

## Abbreviations

A: *Achillea clusiana*
C: *Campanula pulla*
DP: Read depth
E: Eastness
EAA: Environmental-association analysis
ELE: Elevation
*F*_*IS*_: Inbreeding coefficient
*F*_*ST*_: Fixation index
GBS: Genotyping-by-sequencing
GQ: Genotype quality
GTRCAT: General time reversible model incl. rate heterogeneity across sites
H: Hochschwab
*H*_I_: Observed heterozygosity
*H*_S_: Expected heterozygosity
IBD: Isolation-by-distance
K: Admonter Kaibling
L: Lower elevation
LD: Linkage disequilibrium
LGM: Last glacial maximum
M: Medium elevation
m a.s.l.: Meters above sea level
MAT: Mean annual temperature
MCMC: Markov chain Monte Carlo
N: Northness
*N*_e_: Effective population size
nt: Nucleotides
P: September to May precipitation
PC(A): Principal component (analysis)
PCR: Polymerase chain reaction
prec_roy: Precipitation outside of the vegetation period (September-May)
RAD-seq: Restriction-site associated DNA sequencing
RDA: Redundancy analysis
S: Schneeberg
SD: Standard deviation
SE: Southeast
SNV: Single nucleotide variant
SSE: South-southeast
T: Temperature
Tmax: Max. temperature
Tmean: Average temperature
Tmin: Min. temperature
U: Upper elevation
π: Nucleotide diversity

## References

[1] IPCC. Climate Change 2021: The Physical Science Basis. Contribution of Working Group I to the Sixth Assessment Report of the Intergovernmental Panel on Climate Change. Cambridge Univ Press, Cambridge and New York in press. 10.1017/9781009157896.

[2] Mountain Research Initiative EDW Working Group (2015). Elevation-dependent warming in mountain regions of the world. Nat Clim Chang.

[3] Auer I, Böhm R, Jurkovic A, Lipa W, Orlik A, Potzmann R, Nieplova E (2007). HISTALP – historical instrumental climatological surface time series of the Greater Alpine Region. Int J Climatol.

[4] Böhm R, Auer I, Brunetti M, Maugeri M, Nanni T, Schöner W (2001). Regional temperature variability in the European Alps: 1760–1998 from homogenized instrumental time series. Int J Climatol.

[5] Beniston M. Climatic change in mountain regions: A review of possible impacts. Clim Change. 2003;59:5–31.

[6] Parmesan C (2006). Ecological and evolutionary responses to recent climate change. Annu Rev Ecol Evol Syst.

[7] Parmesan C, Hanley ME (2015). Plants and climate change: complexities and surprises. Ann Bot.

[8] Walther G-R, Post E, Convey P, Menzel A, Parmesan C, Beebee T (2002). Ecological responses to recent climate change. Nature.

[9] Holt RD (1990). The microevolutionary consequences of climate change. Trends Ecol Evol.

[10] Jump AS, Peñuelas J (2005). Running to stand still: adaptation and the response of plants to rapid climate change. Ecol Lett.

[11] Walther G-R, Beißner S, Burga CA (2005). Trends in the upward shift of alpine plants. J Veg Sci.

[12] Lenoir J, Svenning J (2015). Climate-related range shifts – a global multidimensional synthesis and new research directions. Ecography.

[13] Rumpf SB, Hülber K, Zimmermann NE, Dullinger S (2019). Elevational rear edges shifted at least as much as leading edges over the last century. Glob Ecol Biogeogr.

[14] Körner C, Hiltbrunner E (2021). Why is the alpine flora comparatively robust against climatic warming?. Diversity.

[15] Jay F, Manel S, Alvarez N, Durang E, Thuiller W, Holderegger R (2012). Forecasting changes in population genetic structure of alpine plants in response to global warming. Mol Ecol.

[16] Price TD, Qvarnström A, Irwin DE (2003). The role of phenotypic plasticity in driving genetic evolution. Proc R Soc London.

[17] Franks SJ, Sim S, Weis AE (2007). Rapid evolution of flowering time by an annual plant in response to a climate fluctuation. P Natl Acad Sci USA.

[18] Sun Y, Bossdorf O, Grados RD, Liao Z, Müller-Schärer H (2020). Rapid genomic and phenotypic change in response to climate warming in a widespread plant invader. Glob Chang Biol.

[19] Jump A, Hunt J, Martínez-Izquierdo J, Peñuelas J (2006). Natural selection and climate change: temperature-linked spatial and temporal trends in gene frequency in Fagus sylvatica. Mol Ecol.

[20] Fischer MC, Rellstab C, Tedder A, Zoller S (2013). Population genomic footprints of selection and associations with climate in natural populations of Arabidopsis halleri from the Alps. Mol Ecol.

[21] Fournier-Level A, Korte A, Cooper M, Nordborg M, Schmitt J, Wilczek A (2011). A map of local adaptation in Arabidopsis thaliana. Science.

[22] Anderson JT, Song BH (2020). Plant adaptation to climate change — Where are we?. J Syst Evol.

[23] Franks SJ, Hoffmann AA (2012). Genetics of climate change adaptation. Annu Rev Genet.

[24] Sexton JP, Mcintyre PJ, Angert AL, Rice KJ (2009). Evolution and ecology of species range limits. Annu Rev Ecol Evol Syst.

[25] Kawecki TJ, Lenski RE, Ebert D, Hollis B, Olivieri I, Whitlock MC (2012). Experimental evolution. Trends Ecol Evol.

[26] Angert AL, Bontrager MG, Ågren J (2020). What do we really know about adaptation at range edges ?. Annu Rev Ecol Evol Syst.

[27] Eckert C, Samis K, Lougheed S (2008). Genetic variation across species’ geographical ranges: the central – marginal hypothesis and beyond. Mol Ecol.

[28] Barton N (2001). Adaptation at the edge of a species’ range. Integrating Ecology and Evolution in a Spatial Context.

[29] Garant D, Forde SE, Hendry AP (2007). The multifarious effects of dispersal and gene flow on contemporary adaptation. Funct Ecol.

[30] Case TJ, Taper ML (2000). Interspecific competition, environmental gradients, gene flow, and the coevolution of species’ borders. Am Nat.

[31] Kirkpatrick M, Barton NH (1997). Evolution of a species’ range. Am Nat.

[32] Antonovics J (1976). The nature of limits to natural selection. Ann Missouri Bot Gard.

[33] Bridle JR, Vines TH (2006). Limits to evolution at range margins: when and why does adaptation fail?. Trends Ecol Evol.

[34] Wolkovich EM, Cook BI, Allen JM, Crimmins TM, Betancourt JL, Travers SE (2012). Warming experiments underpredict plant phenological responses to climate change. Nature.

[35] De Frenne P, Graae BJ, Rodríguez-Sánchez F, Kolb A, Chabrerie O, Decocq G (2013). Latitudinal gradients as natural laboratories to infer species’ responses to temperature. J Ecol.

[36] Körner C (2007). The use of “altitude” in ecological research. Trends Ecol Evol.

[37] Honnay O, Verheyen K, Butaye J, Jacquemyn H, Bossuyt B, Hermy M (2002). Possible effects of habitat fragmentation and climate change on the range of forest plant species. Ecol Lett.

[38] Bender O, Borsdorf A, Fischer A, Stotter J. Mountains under climate and global change conditions – research results in the Alps. In: Climate Change - Geophysical Foundations and Ecological Effects. BoD–Books on Demand. 2011. p. 403–22. 10.5772/24574.

[39] Hewitt G (2000). The genetic legacy of the Quaternary ice ages. Nature.

[40] Schönswetter P, Stehlik I, Holderegger R, Tribsch A (2005). Molecular evidence for glacial refugia of mountain plants in the European Alps. Mol Ecol.

[41] Tribsch A, Schönswetter P (2003). Patterns of endemism and comparative phylogeography confirm palaeoenvironmental evidence for pleistocene refugia in the Eastern Alps. Taxon.

[42] Paun O, Schönswetter P, Winkler M, Tribsch A (2008). Historical divergence vs. contemporary gene flow: Evolutionary history of the calcicole Ranunculus alpestris group (Ranunculaceae) in the European Alps and the Carpathians. Mol Ecol.

[43] Winkler M, Tribsch A, Paun O, Englisch T, Schönswetter P (2010). Pleistocene distribution range shifts were accompanied by breeding system divergence within Hornungia alpina (Brassicaceae) in the Alps. Mol Phylogenet Evol.

[44] Dullinger S, Mang T, Dirnböck T, Ertl S, Gattringer A, Grabherr G (2011). Patch configuration affects alpine plant distribution. Ecography.

[45] Dedieu J-P, Randin C, Zappa M, Dullinger S. Modelling the effect of changing snow cover regimes on alpine plant species distribution in Alpine context. A merging of theory and practice. Proceedings of the International Snow Science Workshop, Grenoble, France, October 7–11 2013, 2013.

[46] Blois JL, Williams JW, Fitzpatrick MC, Jackson ST, Ferrier S (2013). Space can substitute for time in predicting climate-change effects on biodiversity. P Natl Acad Sci USA.

[47] CHELSA Data n.d. https://chelsa-climate.org/ (accessed 13 Sept, 2022).

[48] Elshire RJ, Glaubitz JC, Sun Q, Poland JA, Kawamoto K, Buckler ES (2011). A robust, simple genotyping-by-sequencing (GBS) approach for high diversity species. PLoS One.

[49] Baird NA, Etter PD, Atwood TS, Currey MC, Shiver AL, Lewis ZA (2008). Rapid SNP discovery and genetic mapping using sequenced RAD markers. PLoS One.

[50] Weiß CL, Pais M, Cano LM, Kamoun S, Burbano HA (2018). nQuire: A statistical framework for ploidy estimation using next generation sequencing. BMC Bioinform.

[51] Gadella T (1964). Cytotaxonomic studies in the genus Campanula. Wentia.

[52] Dobeś C, Vitek E (2001). Documented chromosome number checklist of Austrian vascular plants. Taxon.

[53] Ehrendorfer F, Guo Y-P (2006). Multidisciplinary studies on Achillea sensu lato (Compositae-Anthemideae): new data on systematics and phylogeography. Willdenowia.

[54] Catchen JM, Amores A, Hohenlohe P, Cresko W, Postlethwait JH (2011). Stacks: Building and genotyping loci de novo from short-read sequences. G3: Genes Genome Genet.

[55] Hohenlohe PA, Bassham S, Etter PD, Stiffler N, Johnson EA, Cresko WA (2010). Population genomics of parallel adaptation in threespine stickleback using sequenced RAD tags. PLoS Genet.

[56] Kalinowski ST (2005). HP-RARE 1.0: A computer program for performing rarefaction on measures of allelic richness. Mol Ecol Notes.

[57] Stamatakis A (2014). RAxML version 8: A tool for phylogenetic analysis and post-analysis of large phylogenies. Bioinformatics.

[58] Pritchard JK, Stephens M, Donnelly P (2000). Inference of population structure using multilocus genotype data. Genetics.

[59] Falush D, Stephens M, Pritchard JK (2003). Inference of population structure using multilocus genotype data: linked loci and correlated allele frequencies. Genetics.

[60] Evanno G, Regnaut S, Goudet J (2005). Detecting the number of clusters of individuals using the software STRUCTURE: A simulation study. Mol Ecol.

[61] Kopelman N, Mayzel J, Jakobsson M, Rosenberg N, Mayrose I (2015). CLUMPAK: a program for identifying clustering modes and packaging population structure inferences across K. Mol Ecol Resour.

[62] Forester BR, Lasky JR, Wagner HH, Urban DL (2018). Comparing methods for detecting multilocus adaptation with multivariate genotype – environment associations. Mol Ecol.

[63] Stöcklin J, Kuss P, Pluess AR (2009). Genetic diversity, phenotypic variation and local adaptation in the alpine landscape: case studies with alpine plant species. Bot Helv.

[64] De VP, Mouterde M, Gaggiotti OE, Till-Bottraud I (2018). Patterns of phenotypic plasticity and local adaptation in the wide elevation range of the alpine plant Arabis alpina. J Ecol.

[65] Reisch C, Rosbakh S (2021). Patterns of genetic variation in European plant species depend on altitude. Divers Distrib.

[66] Dirnböck T, Essl F, Rabitsch W (2011). Disproportional risk for habitat loss of high-altitude endemic species under climate change. Glob Chang Biol.

[67] Barry R (2008). Mountain weather and climate.

[68] Winkler M, Lamprecht A, Steinbauer K, Hülber K, Theurillat J-P, Breiner F (2016). The rich sides of mountain summits – a pan-European view on aspect preferences of alpine plants. J Biogeogr.

[69] Lamprecht A, Semenchuk PR, Steinbauer K, Winkler M, Pauli H (2018). Climate change leads to accelerated transformation of high-elevation vegetation in the central Alps. New Phytol.

[70] Hülber K, Wessely J, Gattringer A, Moser D, Kuttner M, Essl F (2016). Uncertainty in predicting range dynamics of endemic alpine plants under climate warming. Glob Chang Biol.

[71] Cotto O, Wessely J, Georges D, Schmid M, Dullinger S (2017). A dynamic eco-evolutionary model predicts slow response of alpine plants to climate warming. Nat Commun.

[72] Wessely J, Gattringer A, Guillaume F, Hülber K, Klonner G, Moser D (2022). Climate warming may increase the frequency of cold-adapted haplotypes in alpine plants. Nat Clim Chang.

[73] Gonzalo-Turpin H, Hazard L (2009). Local adaptation occurs along altitudinal gradient despite the existence of gene flow in the alpine plant species Festuca eskia. J Ecol.

[74] Alvarez N, Thiel-Egenter C, Holderegger R, Manel S, Schönswetter P, Taberlet P (2009). History or ecology ? Substrate type as a major driver of spatial genetic structure in Alpine plants. Ecol Lett.

[75] Fischer MA, Oswald K, Adler W. Exkursionsflora für Österreich, Liechtenstein und Südtirol. 3. Auflage. OÖ Landesmuseen, Land Oberösterreich, Austria; 2008.

[76] Scheffknecht S, Dullinger S, Grabherr G, Hülber K (2007). Mating systems of snowbed plant species of the northeastern Calcareous Alps of Austria. Acta Oecol.

[77] Karger DN, Conrad O, Böhner J, Kawohl T, Kreft H, Soria-Auza RW (2017). Climatologies at high resolution for the earth’s land surface areas. Sci Data.

[78] Karger DN, Conrad O, Böhner J, Kawohl T, Kreft H, Soria-Auza RW, et al. Data from: Climatologies at high resolution for the earth’s land surface areas. EnviDat 2018. 10.16904/envidat.228.v2.1. 10.1038/sdata.2017.122 PMC5584396 28872642

[79] Zimmermann NE, Yoccoz NG, Edwards TC, Meier ES, Thuiller W, Guisan A (2009). Climatic extremes improve predictions of spatial patterns of tree species. Proc Natl Acad Sci U S A.

[80] Dullinger S, Gattringer A, Thuiller W, Moser D, Zimmermann NE, Guisan A (2012). Extinction debt of high-mountain plants under twenty-first-century climate change. Nat Clim Chang.

[81] Murray KD, Borevitz JO (2018). Axe: Rapid, competitive sequence read demultiplexing using a trie. Bioinformatics.

[82] batch_trim.pl n.d. https://github.com/Lanilen/GBS-PreProcess (accessed 13 Sept, 2022).

[83] Martin M (2011). Cutadapt removes adapter sequences from high-throughput sequencing reads. EMBnet J..

[84] Andrews S. FastQC: A quality control tool for high throughput sequence data 2010. https://www.bioinformatics.babraham.ac.uk/projects/fastqc/ (accessed 13 Sept, 2022).

[85] Altschul SF, Gish W, Miller W, Myers EW, Lipman DJ (1990). Basic local alignment search tool. J Mol Biol.

[86] Zhang Z, Schwartz S, Wagner L, Miller W (2000). A greedy algorithm for aligning DNA sequences. J Comput Biol.

[87] Li H. Aligning sequence reads, clone sequences and assembly contigs with BWA-MEM. ArXiv:13033997 2013;00:1–3.

[88] Li H (2011). A statistical framework for SNP calling, mutation discovery, association mapping and population genetical parameter estimation from sequencing data. Bioinformatics.

[89] Li H, Handsaker B, Wysoker A, Fennell T, Ruan J, Homer N (2009). The Sequence Alignment / Map format and SAMtools. Bioinformatics.

[90] Bolger AM, Lohse M, Usadel B (2014). Trimmomatic: A flexible trimmer for Illumina sequence data. Bioinformatics.

[91] Paris JR, Stevens JR, Catchen JM (2017). Lost in parameter space: a road map for stacks. Methods Ecol Evol.

[92] Yokoya K, Roberts Av, Mottley J, Lewis R, Brandham PE (2000). Nuclear DNA Amounts in Roses. Ann Bot..

[93] Greilhuber J, Ebert I (1994). Genome size variation in Pisum sativum. Genome.

[94] Patterson N, Price AL, Reich D (2006). Population structure and Eigenanalysis. PLoS Genet.

[95] CLUMPAK n.d. http://clumpak.tau.ac.il/bestK.html (accessed 13 Sept, 2022).

[96] Data and code generated for this study n.d. 10.6084/m9.figshare.20317953.v1.

