# Supplementary figures and images for: Genome-environment associations along elevation gradients in two snowbed species of the North-Eastern Calcareous Alps
Source: BMC Plant Biol. 2023 Apr 19;23:203. doi: 10.1186/s12870-023-04187-x (PMC10114330; doi:10.1186/s12870-023-04187-x)

% of filter1-3 reads left

A

B

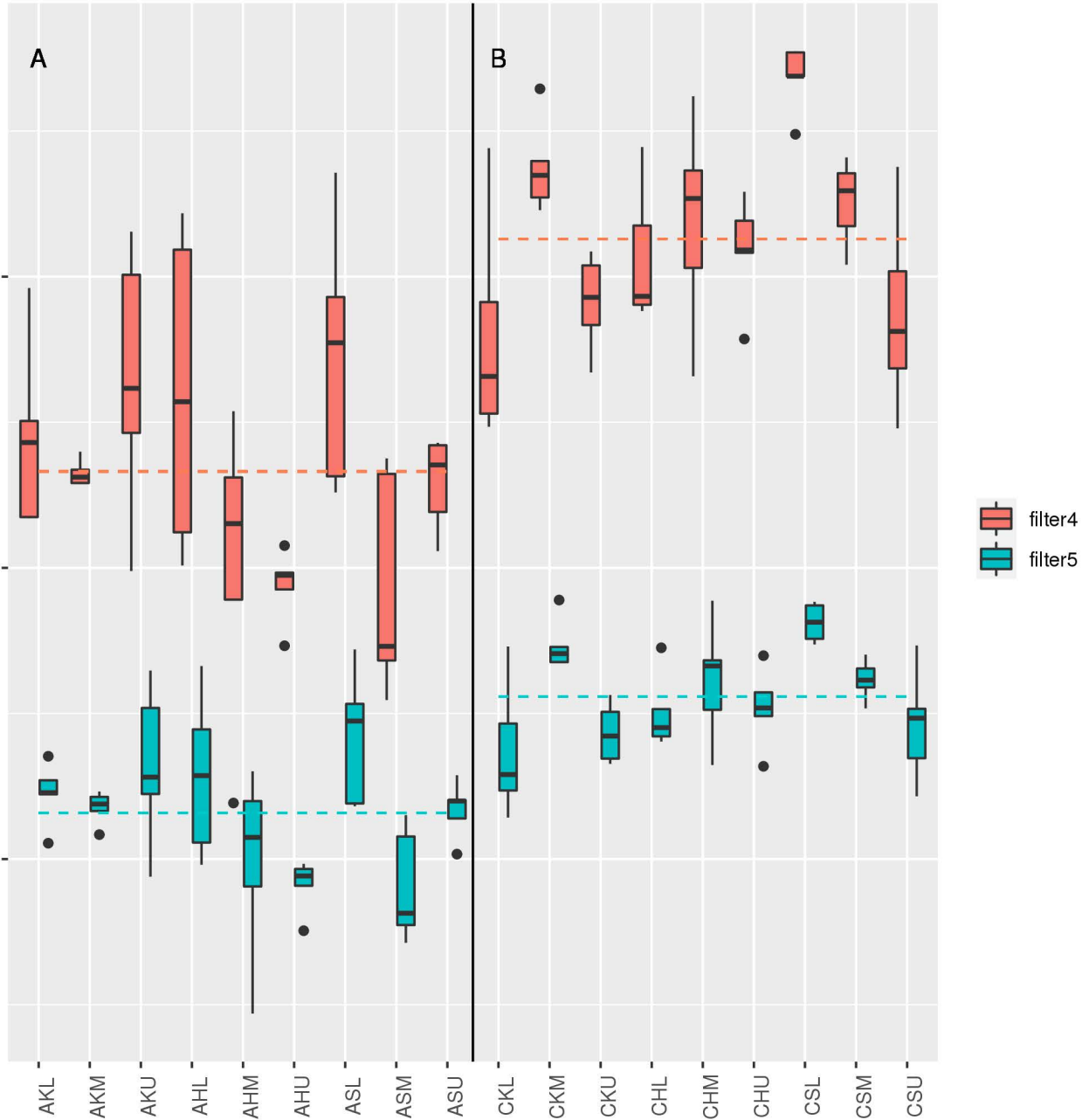

Supplement: Supplementary file 8 — Additional file 8: Figure S1. Percentage of raw reads left after the different filtering steps for samples of each A) Achillea clusiana and B) Campanula pulla population. Filter1-3 corresponds to 100% and is the data after demultiplexing, adapter removal and trimming of CWG overhangs from ApeKI digestion, filter4 is removal of overrepresented sequences and filter5 is trimming to 90 nt. For each species the per-sample average per filtering step is included as dashed line. Population names consist of species (A for A. clusiana, C for C. pulla), mountain (K for Admonter Kaibling, H for Hochschwab, and S for Schneeberg) and elevation (lower L, medium M, and upper U). [file 12870_2023_4187_MOESM8_ESM.pdf]

**A**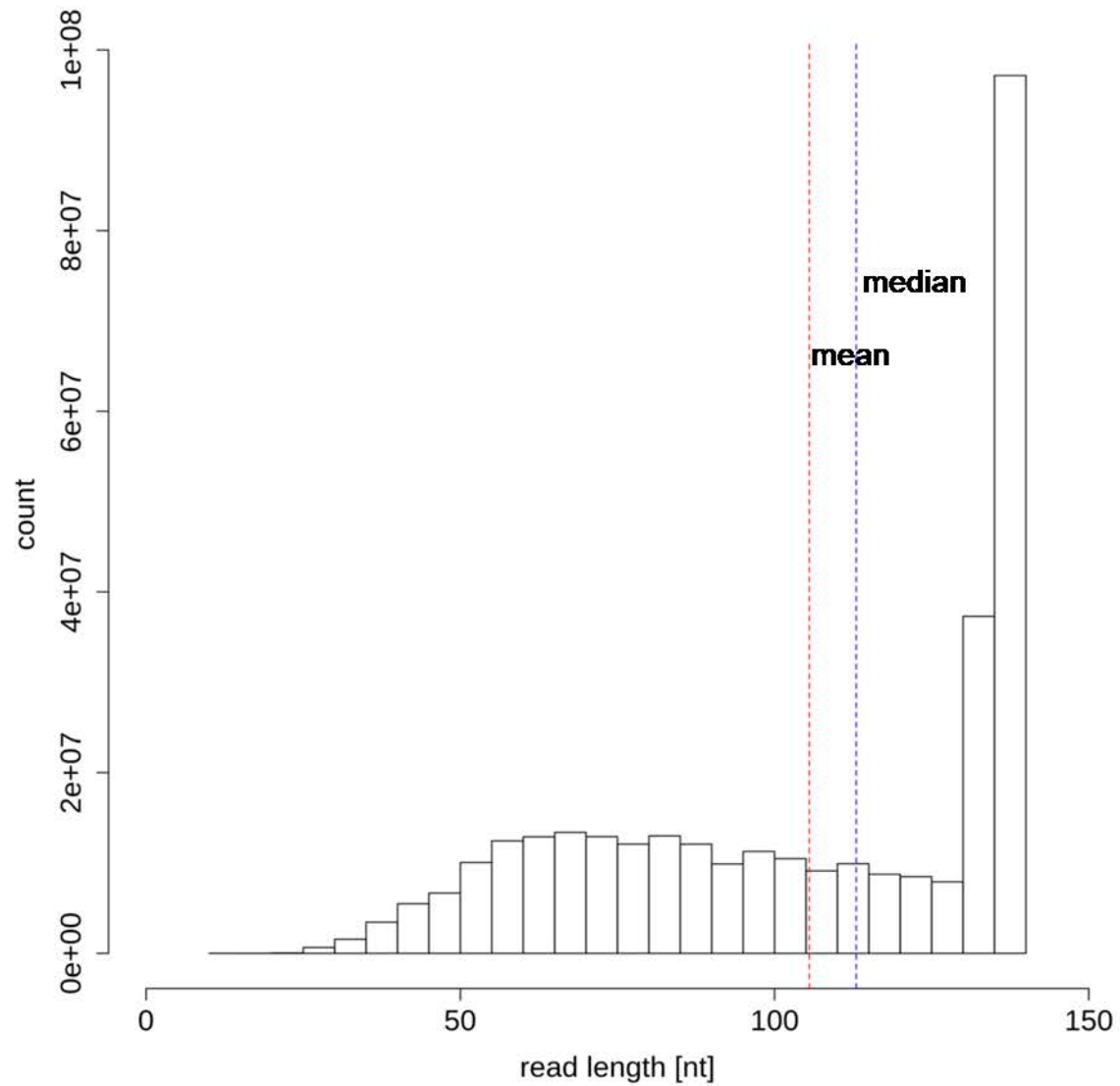**B**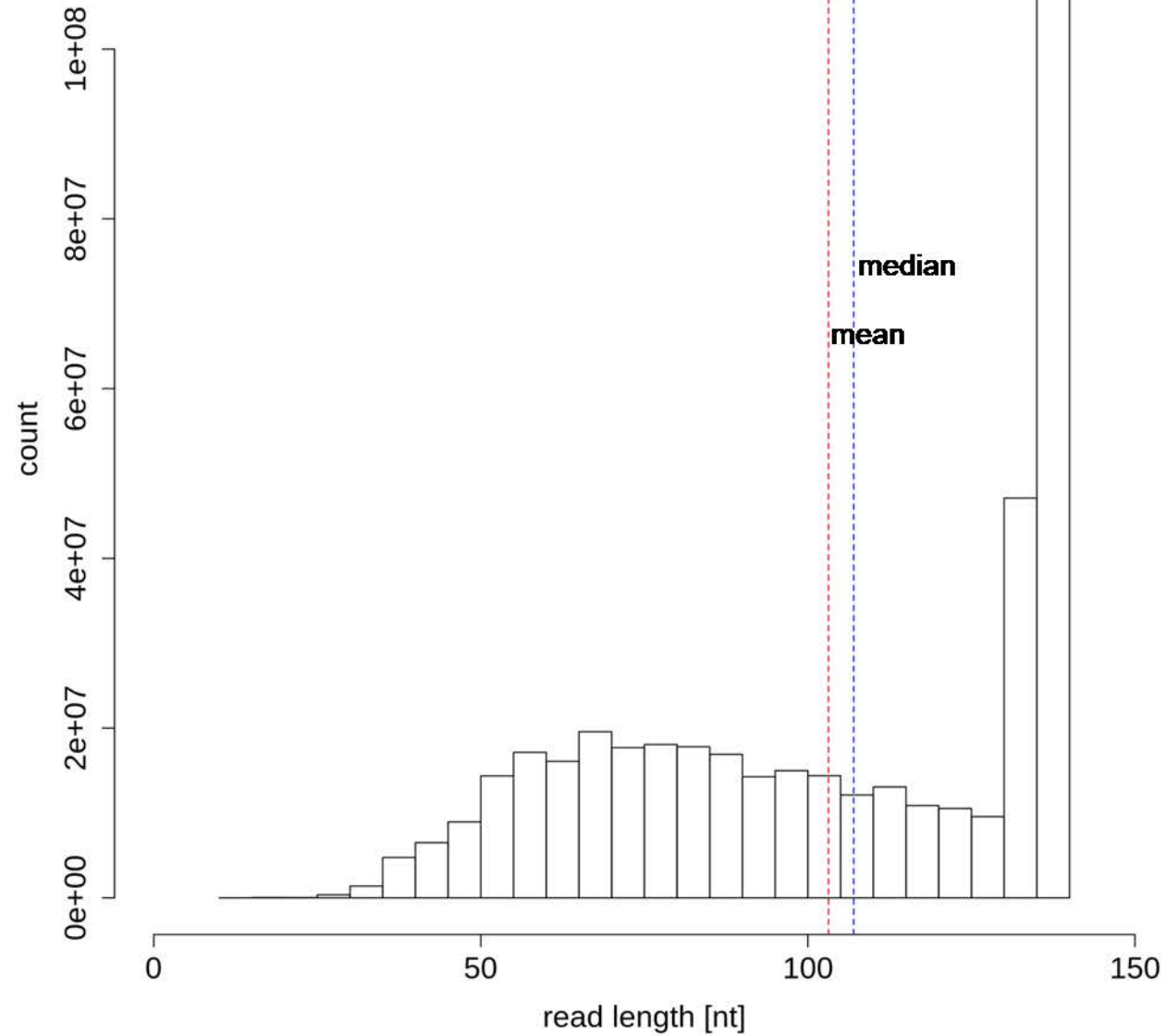

Supplement: Supplementary file 9 — Additional file 9: Figure S2. Distribution of read lengths after applying filter1-3 (demultiplexing, adapter removal and trimming of CWG overhangs from ApeKI digestion) for A) Achillea clusiana and B) for Campanula pulla. Mean and median read lengths are indicated. [file 12870_2023_4187_MOESM9_ESM.pdf]

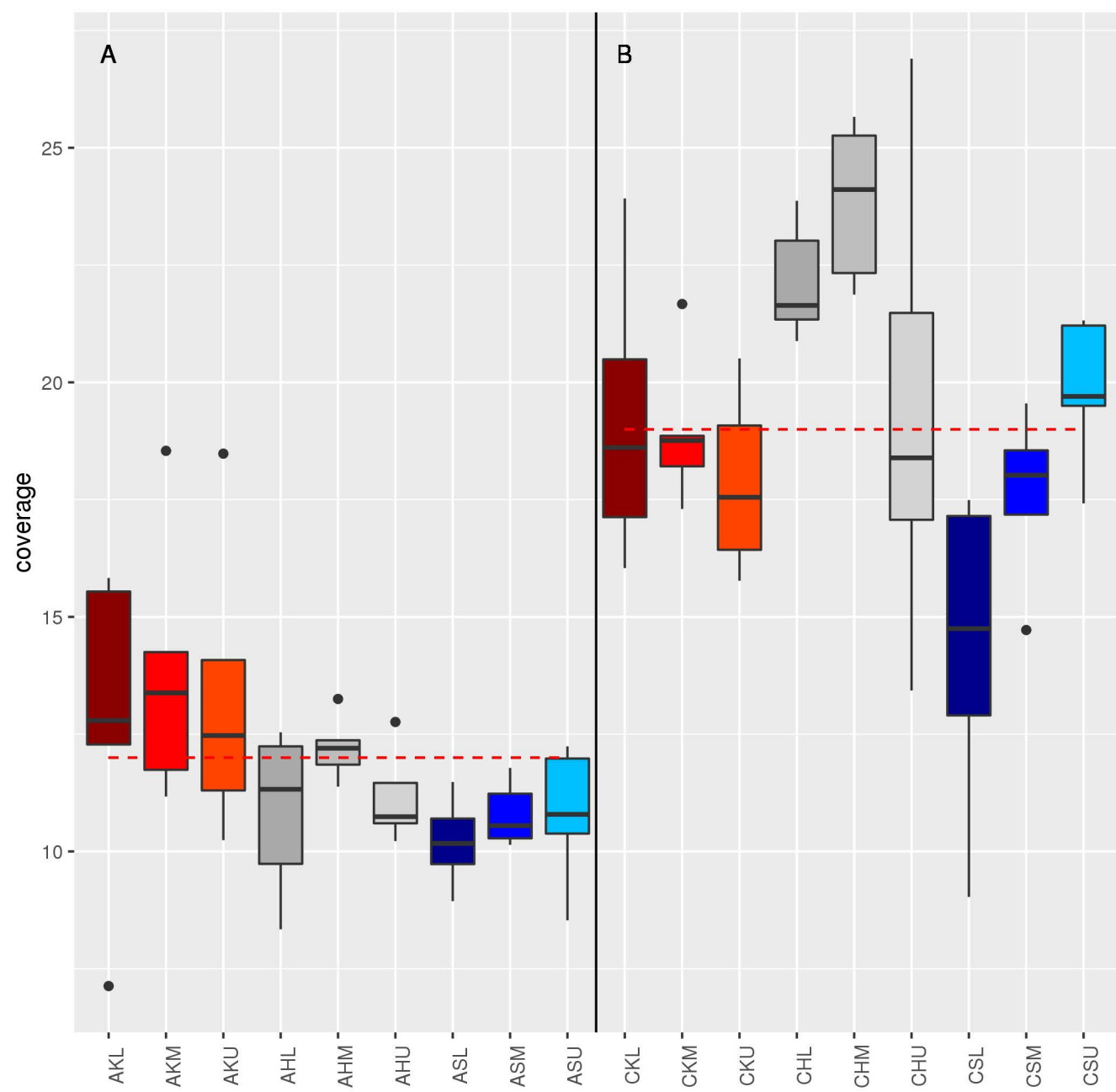

Supplement: Supplementary file 10 — Additional file 10: Figure S3. Stack coverage of stacks per population of A) Achillea clusiana and B) Campanula pulla. Stacks were generated with the ustacks step of the Stacks pipeline. The per-species average coverage is indicated with a dashed red line. Population names consist of species (A for A. clusiana, C for C. pulla), mountain (K for Admonter Kaibling, H for Hochschwab, and S for Schneeberg) and elevation (lower L, medium M, and upper U). [file 12870_2023_4187_MOESM10_ESM.pdf]

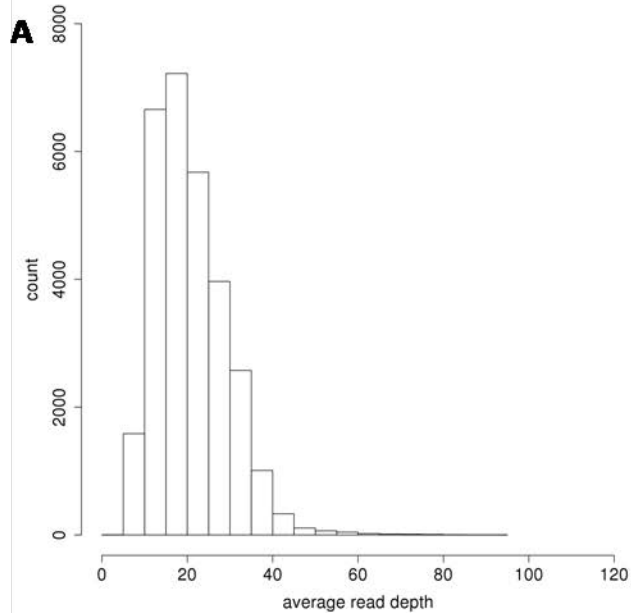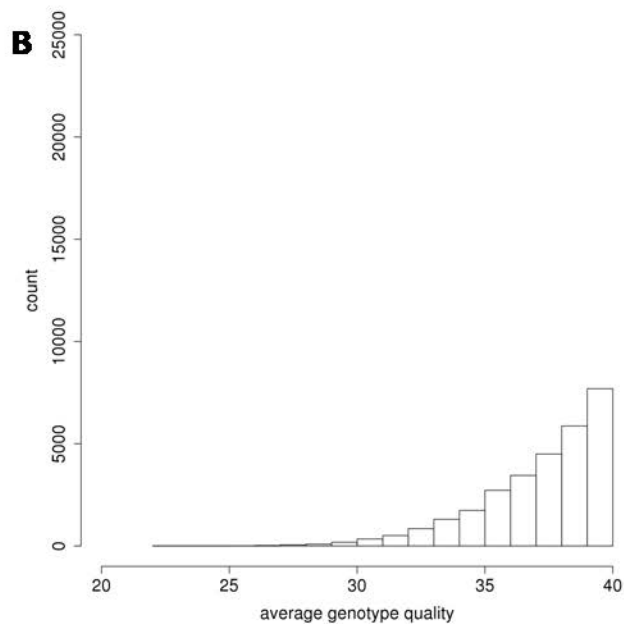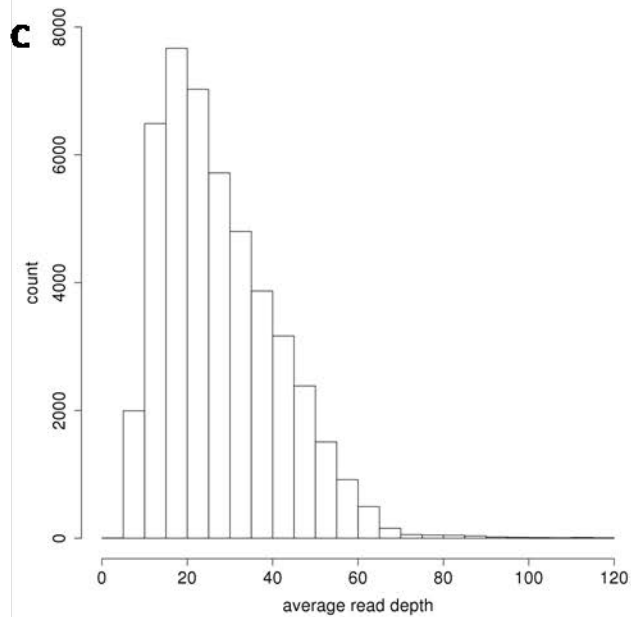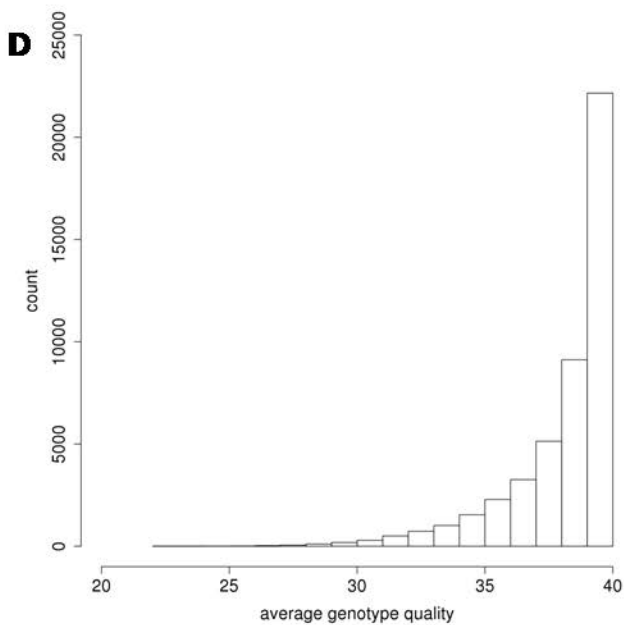

Supplement: Supplementary file 11 — Additional file 11: Figure S4. Distribution of observed stack read depths (DP; A) and C)) and observed genotype qualities (GQ; B) and D)) for the variants detected in Achillea clusiana in the upper panel and Campanula pulla in the lower panel, respectively. Thresholds for genotype quality and read depth filtering of the variants were set per species according to these plots: GQ 32 and DP 4–36 in case of A. clusiana and GQ 33 and DP 4–53 in case of C. pulla, respectively. [file 12870_2023_4187_MOESM11_ESM.pdf]

**A**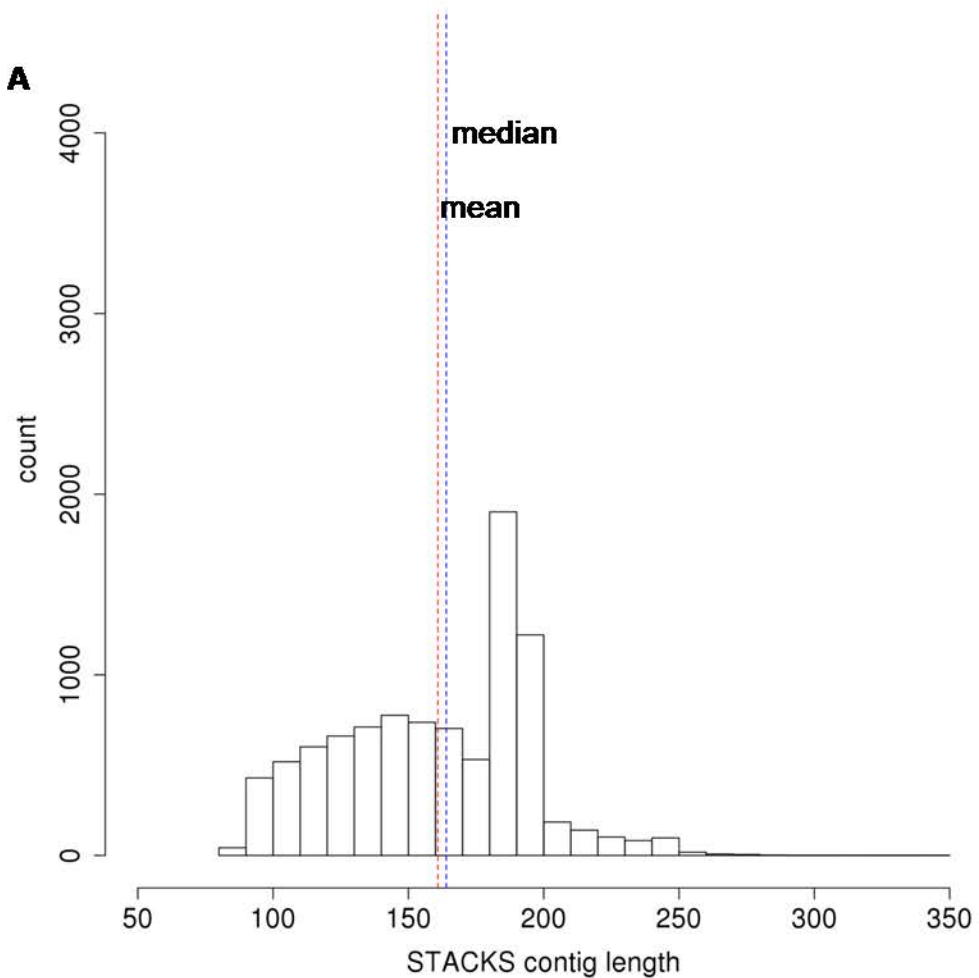**B**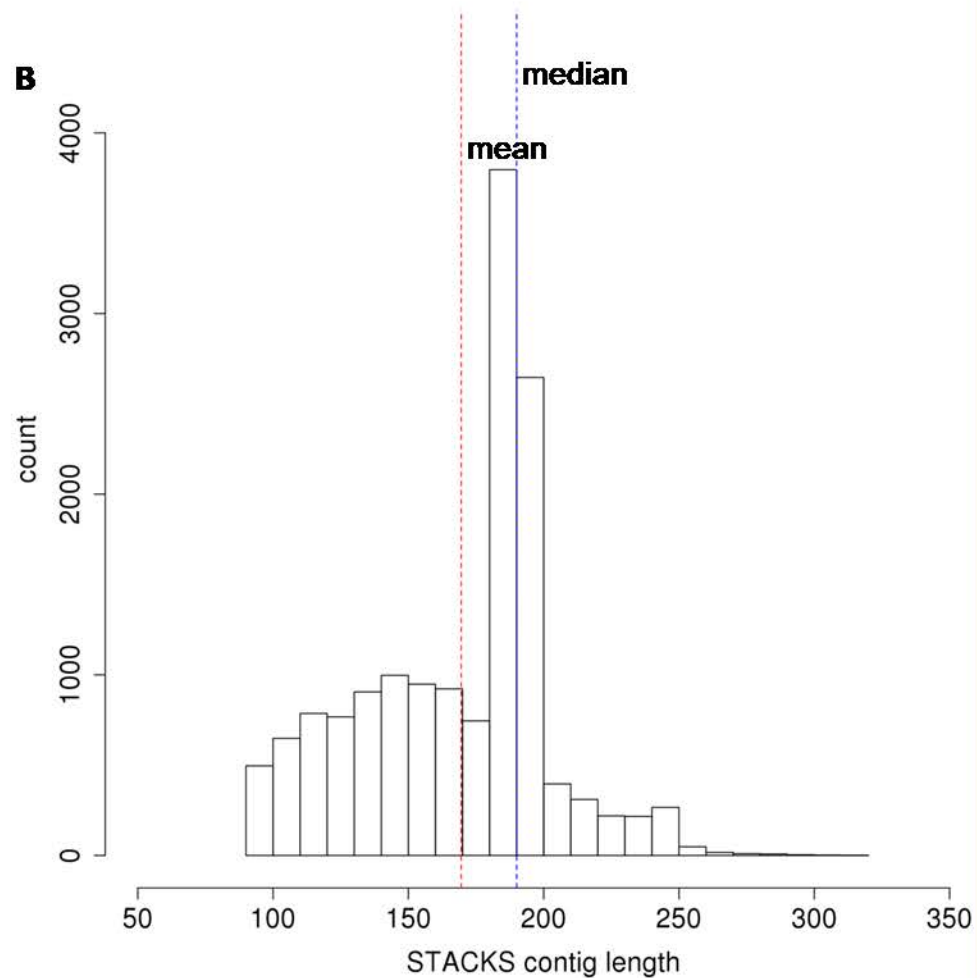

Supplement: Supplementary file 12 — Additional file 12: Figure S5. Length distribution of final quality filtered de novo assembled polymorphic Stacks contigs for Achillea clusiana in A) and for Campanula pulla in B), respectively. Mean and median read lengths are indicated. [file 12870_2023_4187_MOESM12_ESM.pdf]

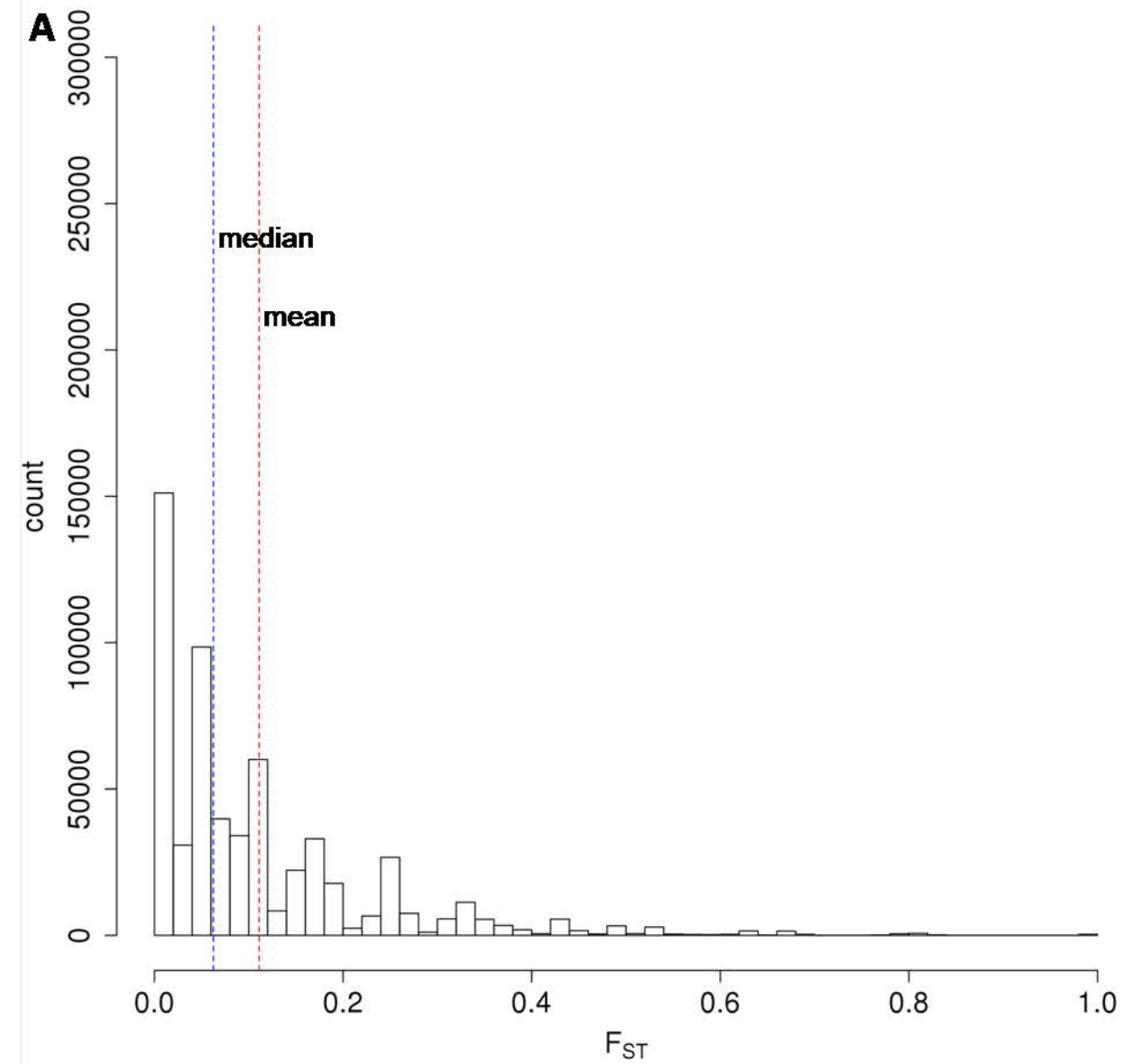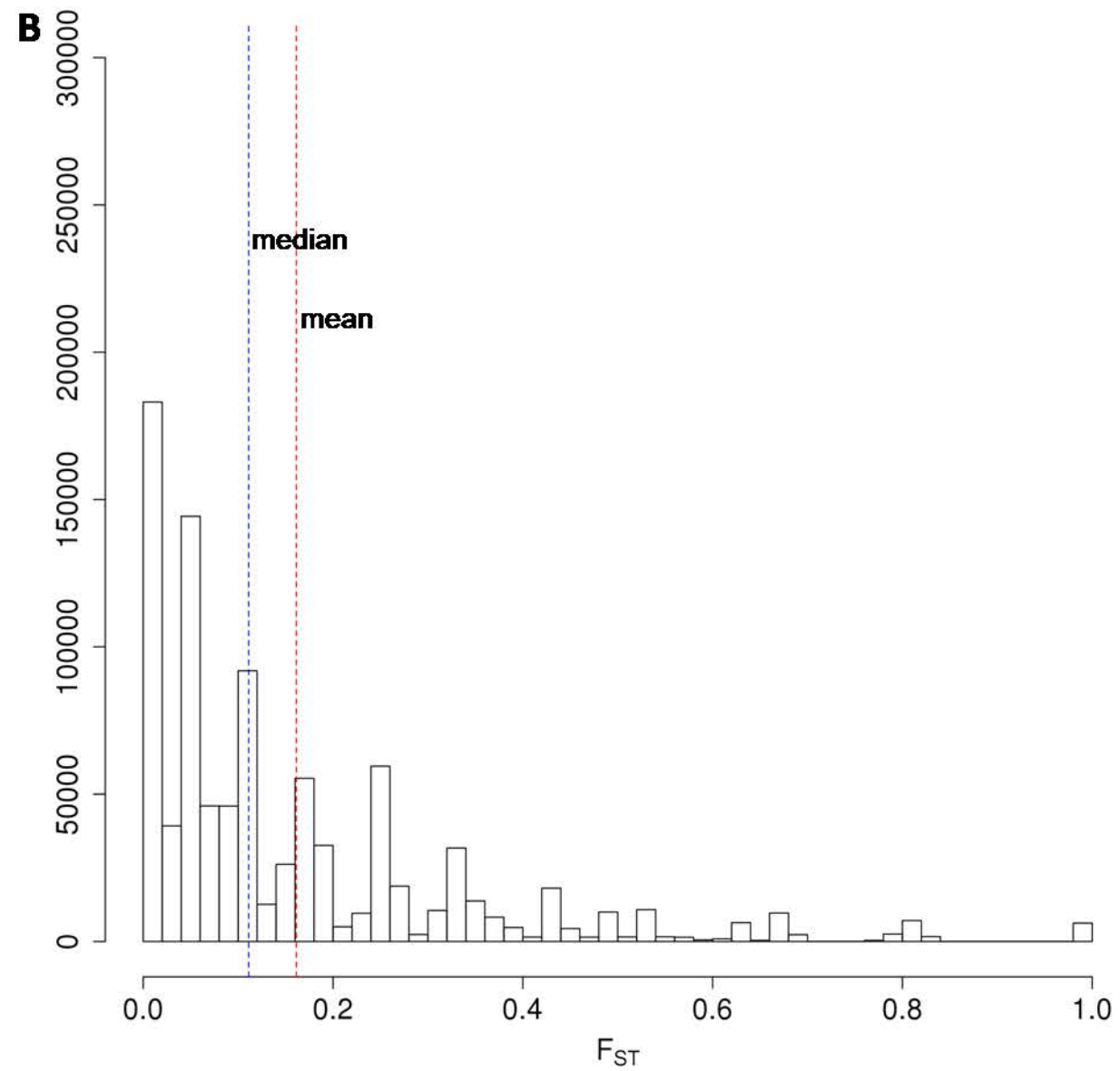

Supplement: Supplementary file 13 — Additional file 13: Figure S6. Distribution of per-site FST values for each quality filtered variant for A) Achillea clusiana and B) Campanula pulla. Mean and median FST values are indicated. [file 12870_2023_4187_MOESM13_ESM.pdf]

**A**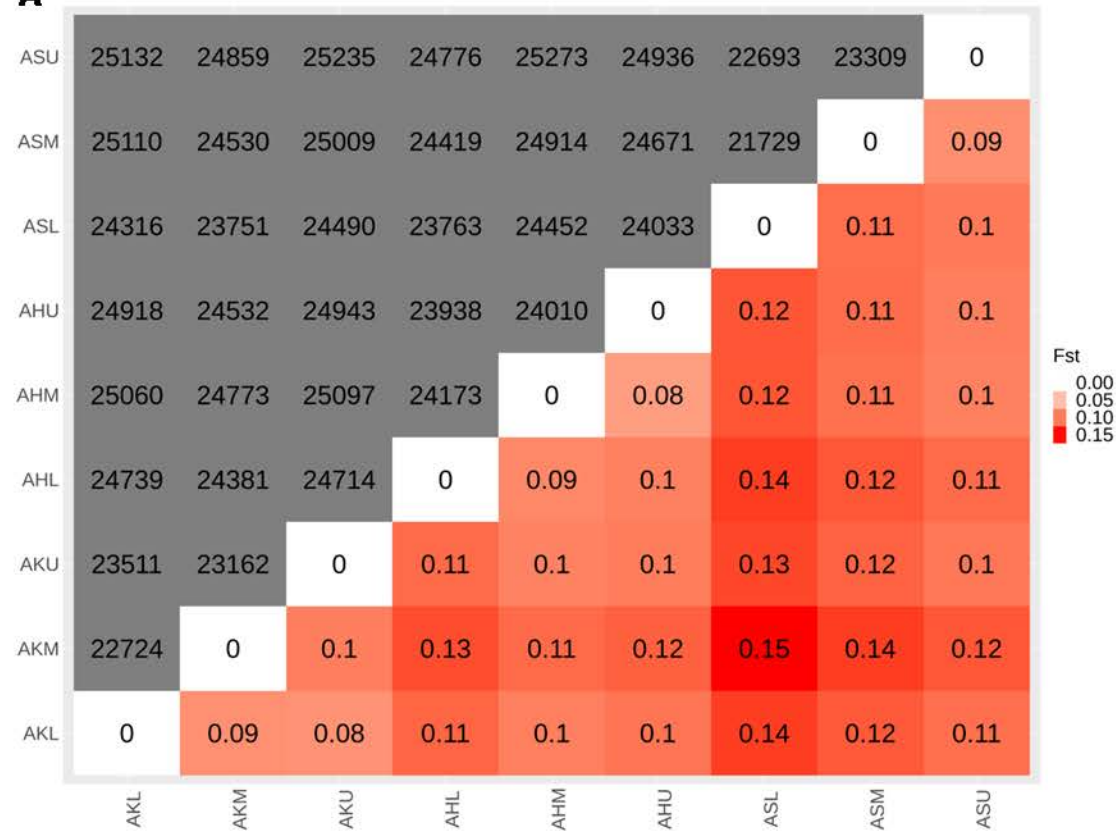**B**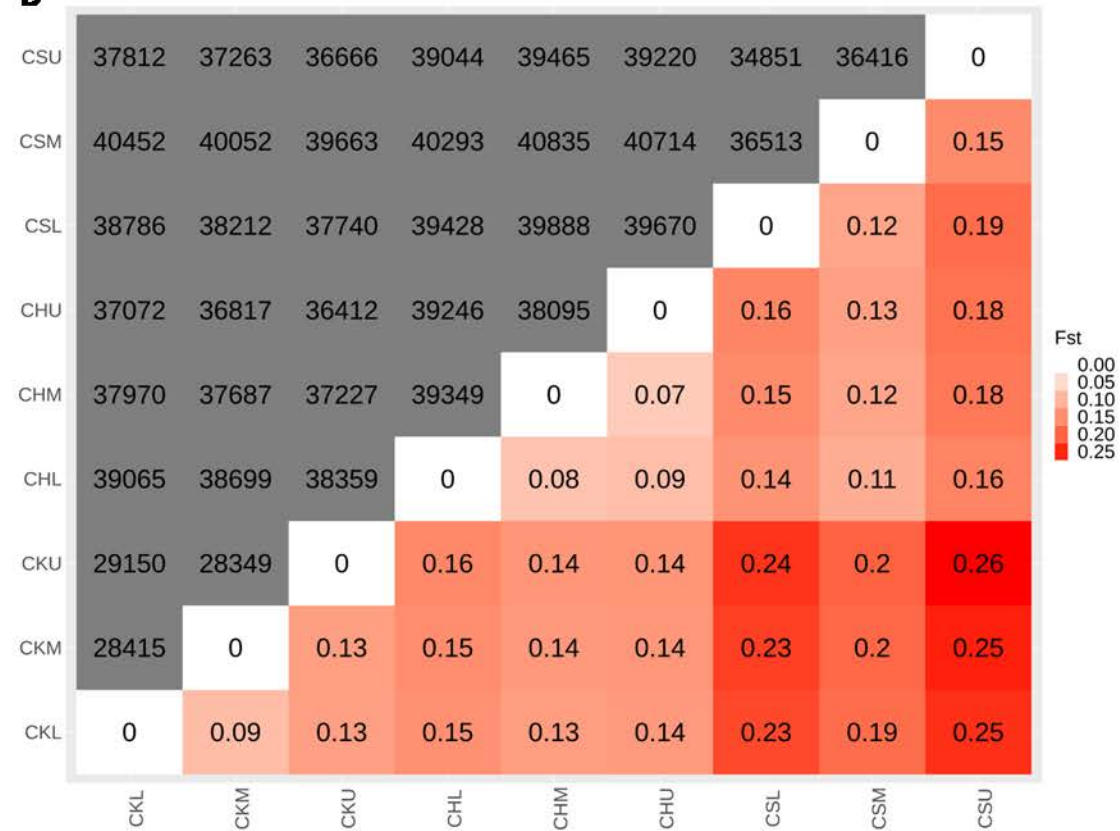

Supplement: Supplementary file 14 — Additional file 14: Figure S7. Pairwise FST values (below the diagonal) for all populations based on variants obtained with Stacks for A) Achillea clusiana and B) for Campanula pulla. Numbers of shared variants are shown in grey above the diagonal. Population names consist of species (A for A. clusiana, C for C. pulla), mountain (K for Admonter Kaibling, H for Hochschwab, and S for Schneeberg) and elevation (lower L, medium M, and upper U). [file 12870_2023_4187_MOESM14_ESM.pdf]

**A**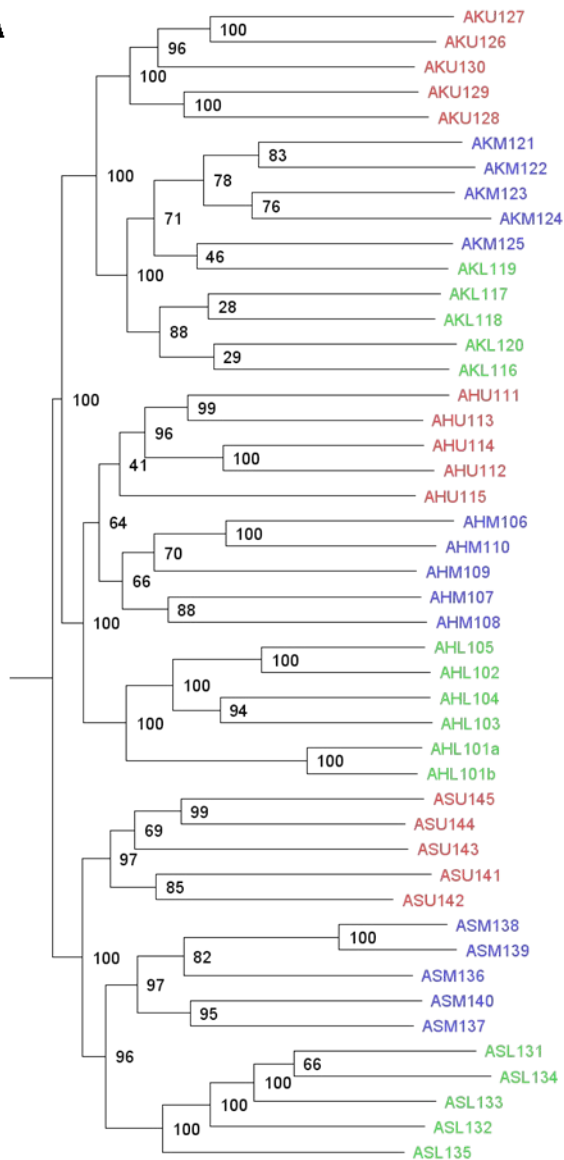**B**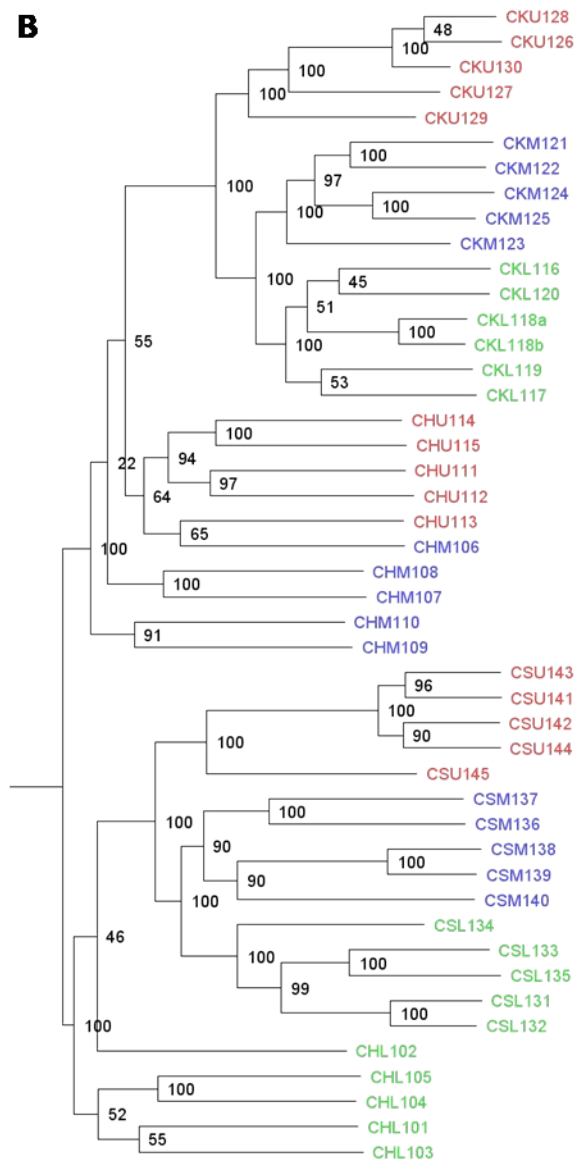

Supplement: Supplementary file 15 — Additional file 15: Figure S8. Midpoint-rooted maximum likelihood tree based on quality filtered variants for A) Achillea clusiana and B) for Campanula pulla. Branches are supported by bootstrap values obtained from 1,000 replicates. Sample names consist of species (A for A. clusiana, C for C. pulla), mountain (K for Admonter Kaibling, H for Hochschwab, and S for Schneeberg), elevation (lower L, medium M, and upper U) and the individual sample ID. [file 12870_2023_4187_MOESM15_ESM.pdf]

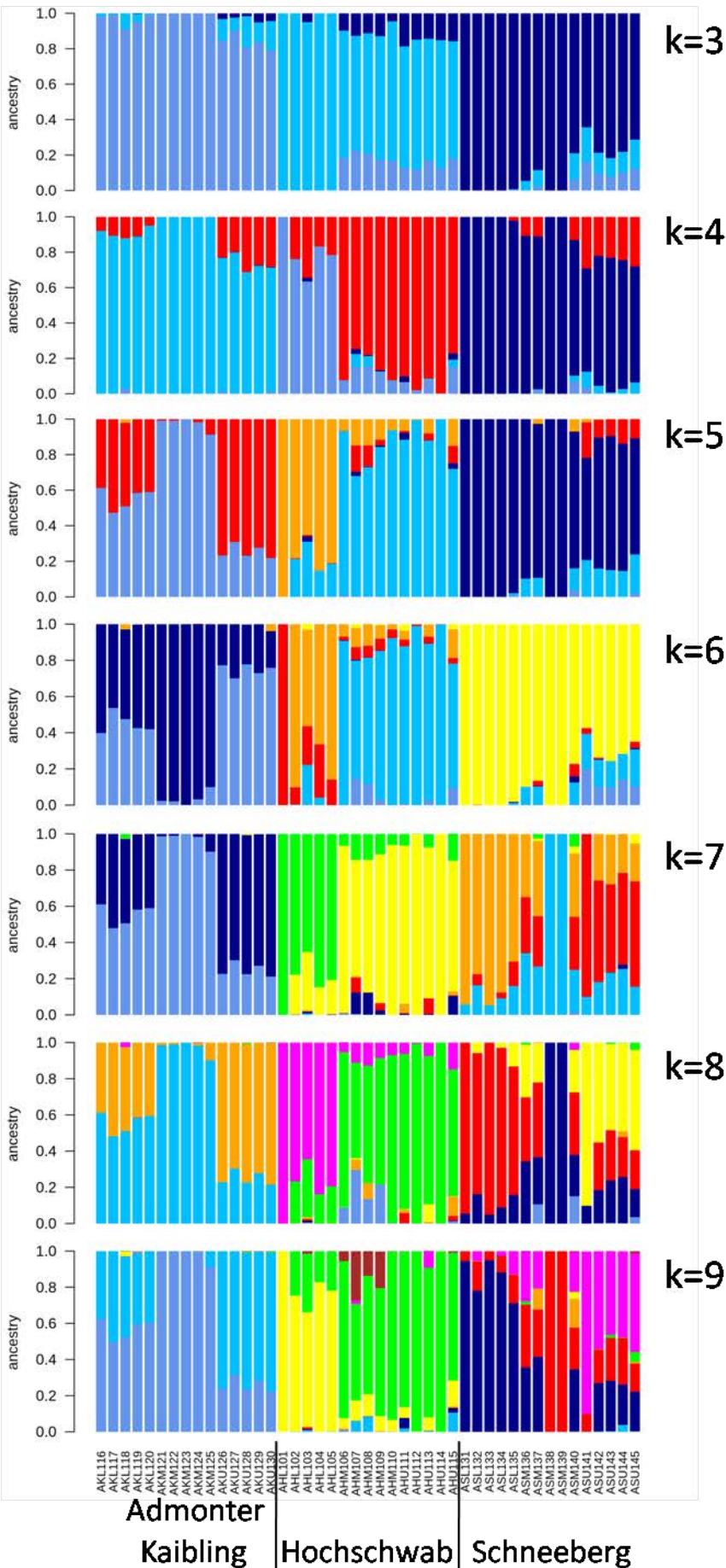

Supplement: Supplementary file 16 — Additional file 16: Figure S9. Admixture results obtained with STRUCTURE assuming k three to nine ancestral populations and using allele frequencies for variants obtained with Stacks for Achillea clusiana. Samples are sorted from left to right starting with the westernmost mountain Admonter Kaibling, over Hochschwab to the easternmost mountain Schneeberg and populations of each mountain are sorted by elevation in increasing order from left to right. Ancestry proportions for each sample are shown on the y-axis. Sample names consist of species (A for A. clusiana), mountain (K for Admonter Kaibling, H for Hochschwab, and S for Schneeberg), elevation (lower L, medium M, and upper U) and the individual sample ID. [file 12870_2023_4187_MOESM16_ESM.pdf]

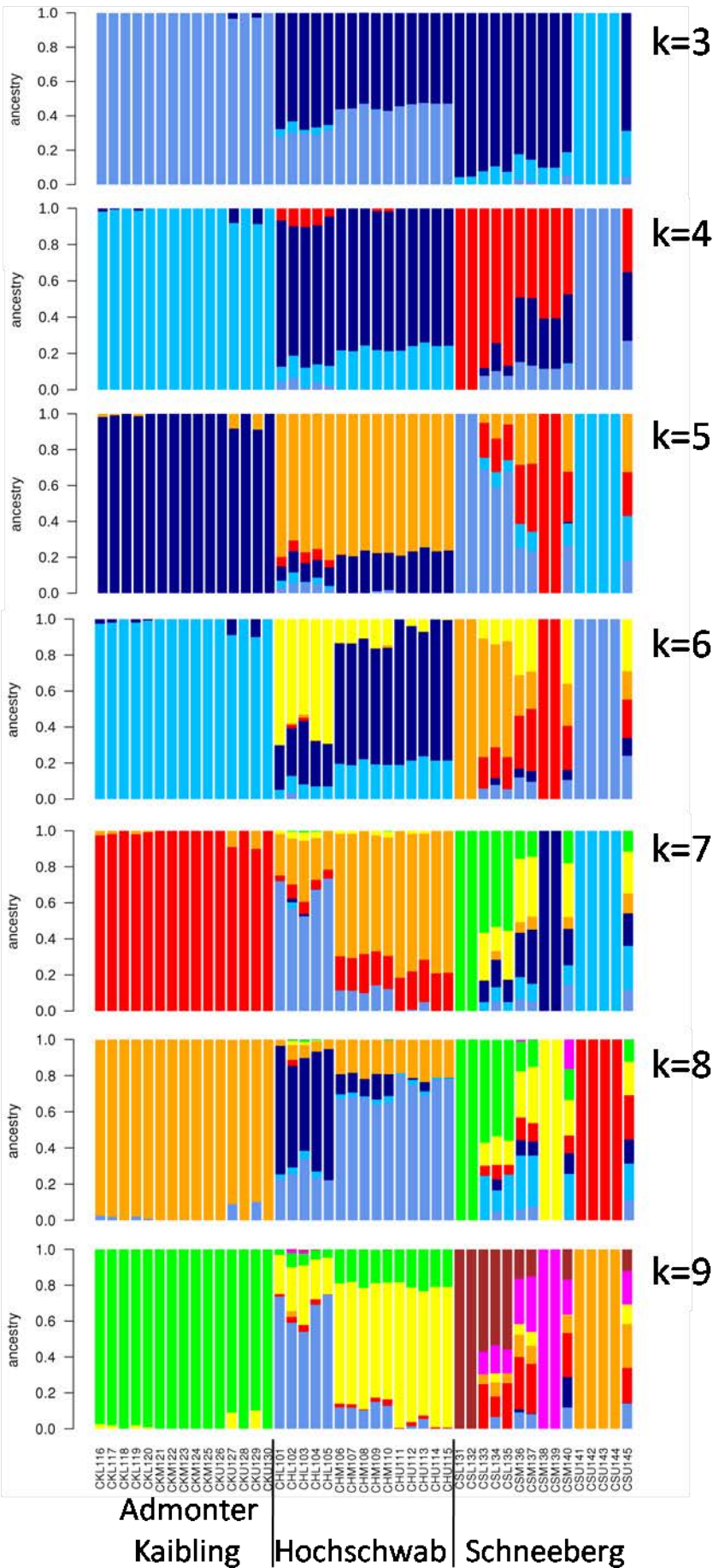

Supplement: Supplementary file 17 — Additional file 17: Figure S10. Admixture results obtained with STRUCTURE assuming k three to nine ancestral populations and using allele frequencies for variants obtained with Stacks for Campanula pulla. Samples are sorted from left to right starting with the westernmost mountain Admonter Kaibling, over Hochschwab to the easternmost mountain Schneeberg and populations of each mountain are sorted by elevation in increasing order from left to right. Ancestry proportions for each sample are shown on the y-axis. Sample names consist of species (C for C. pulla), mountain (K for Admonter Kaibling, H for Hochschwab, and S for Schneeberg), elevation (lower L, medium M, and upper U) and the individual sample ID. [file 12870_2023_4187_MOESM17_ESM.pdf]

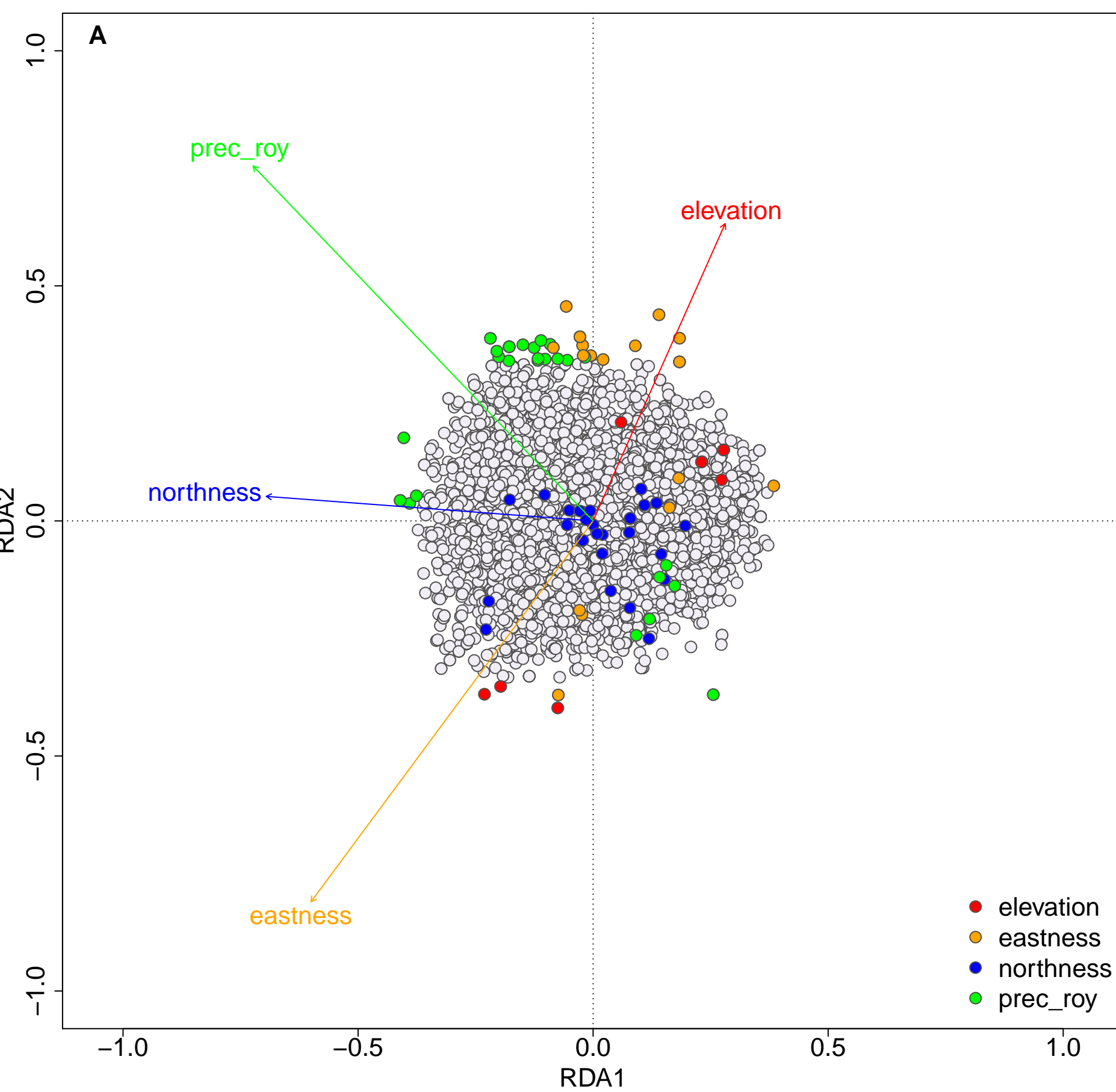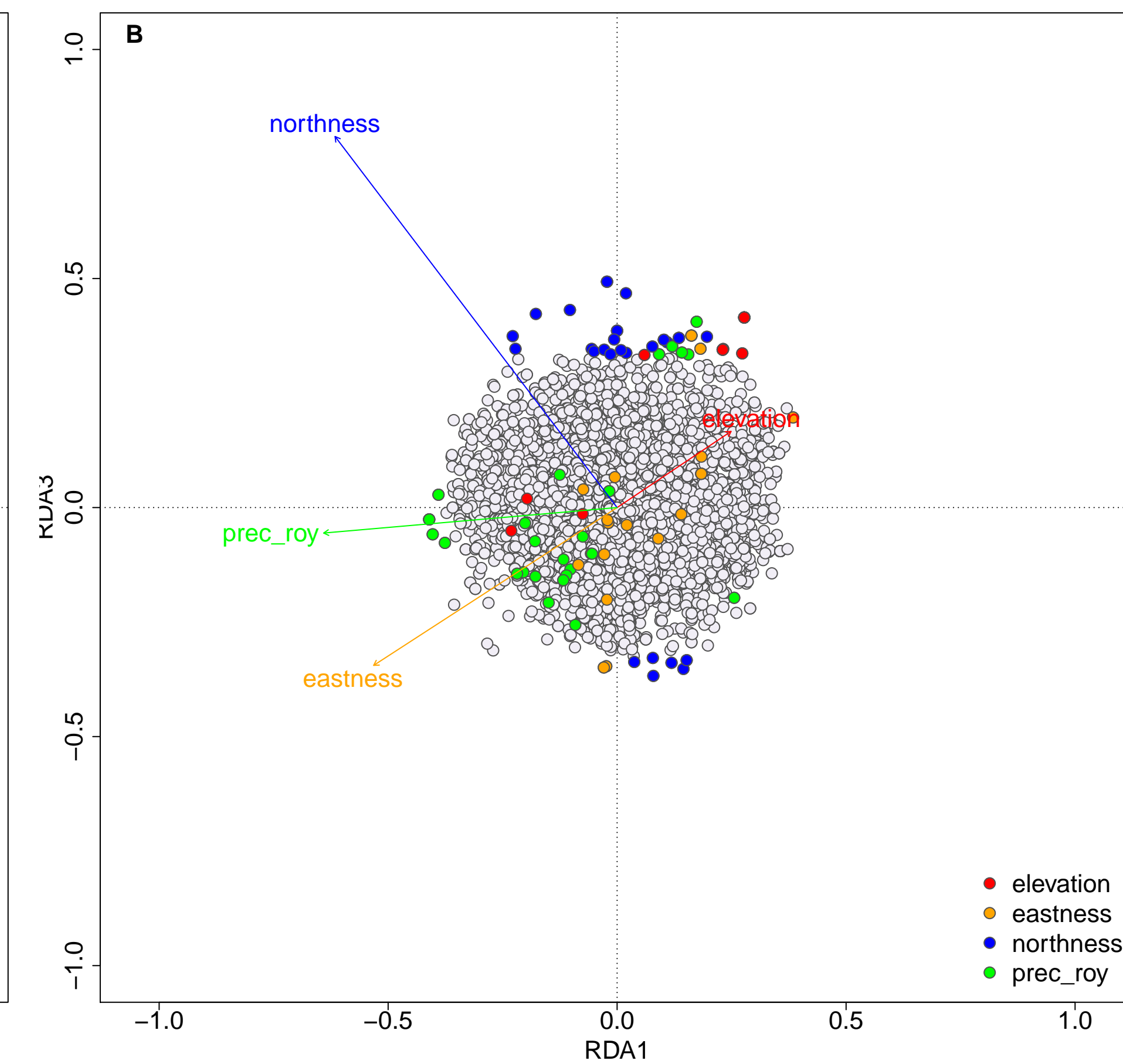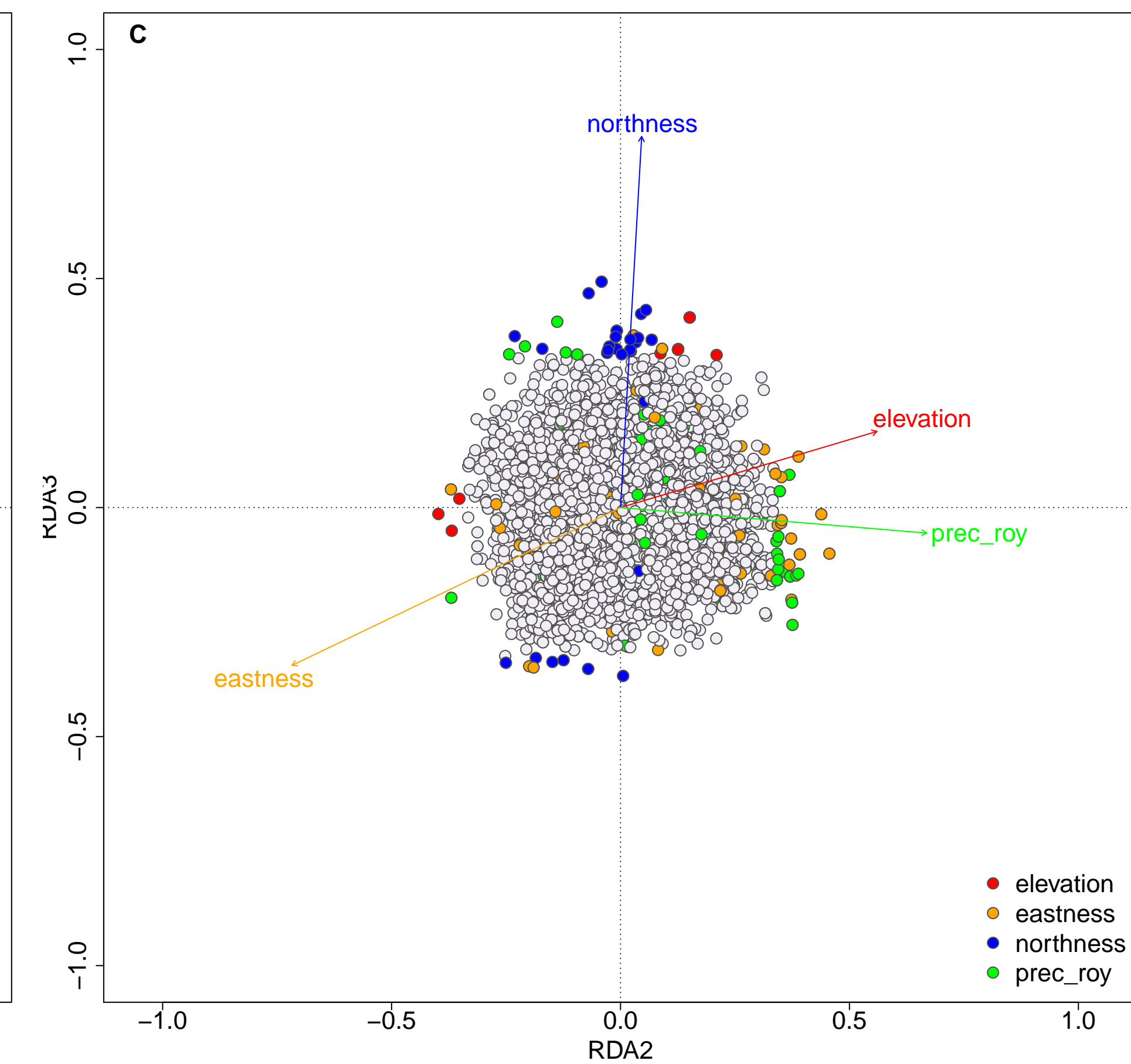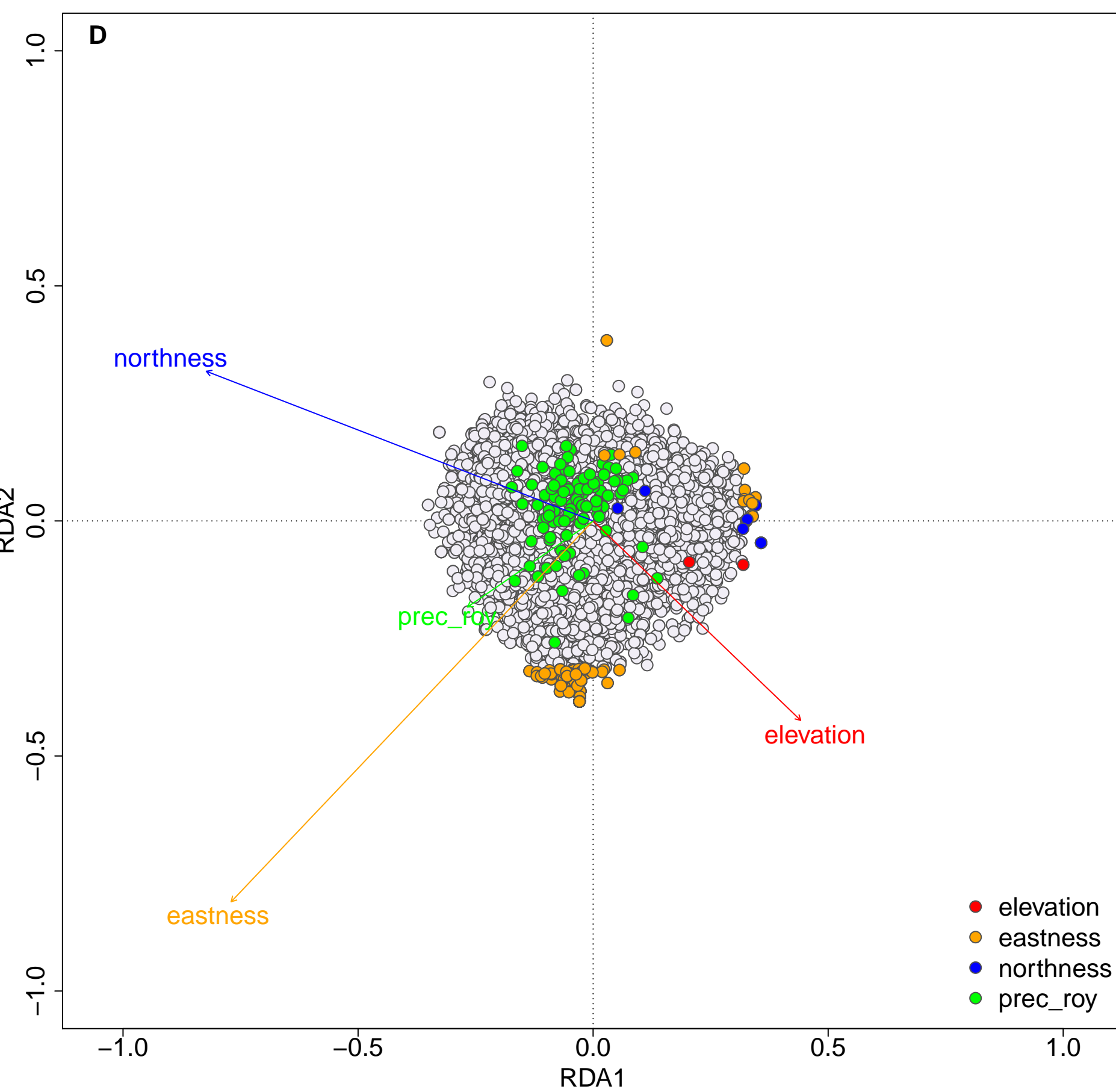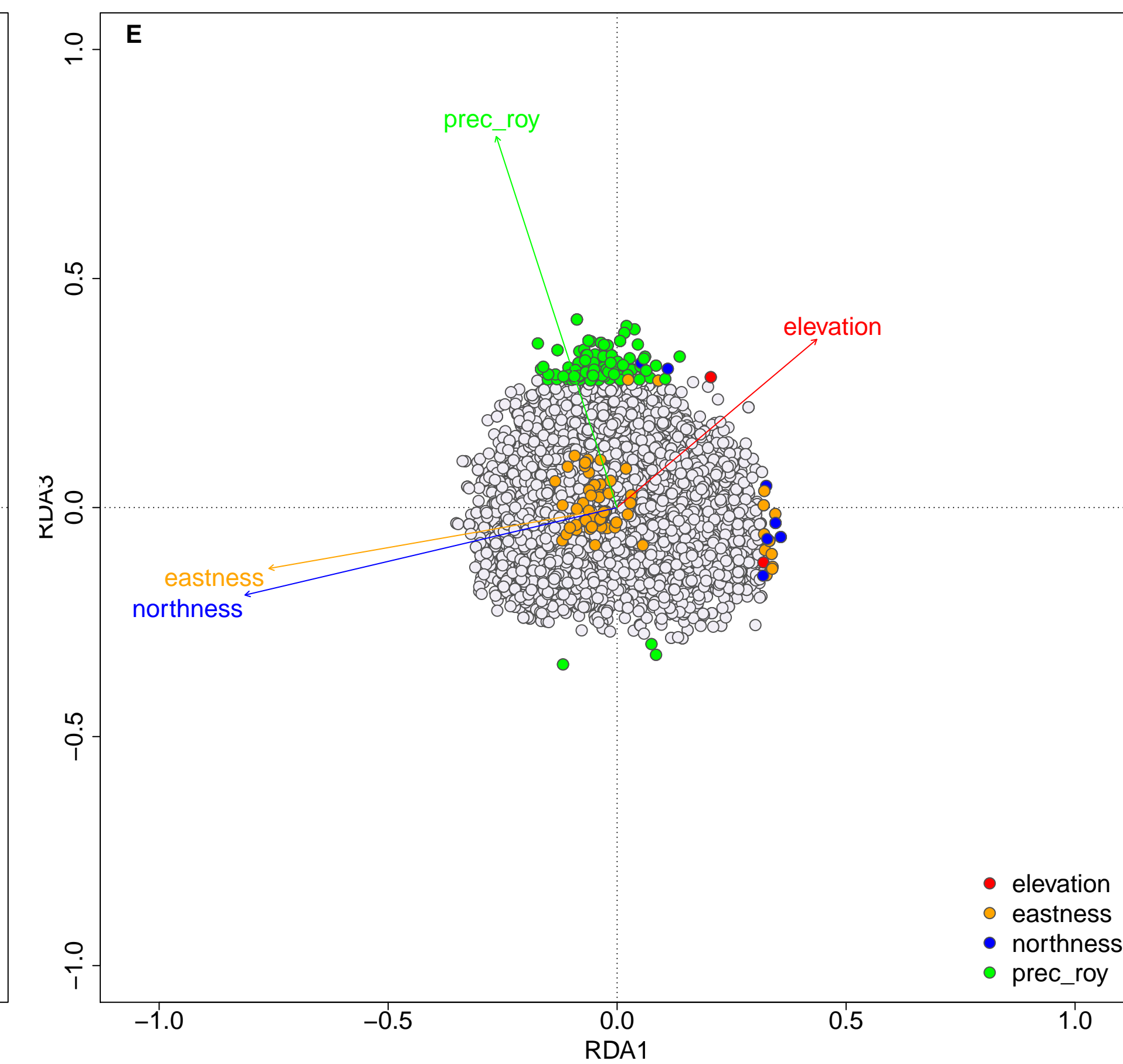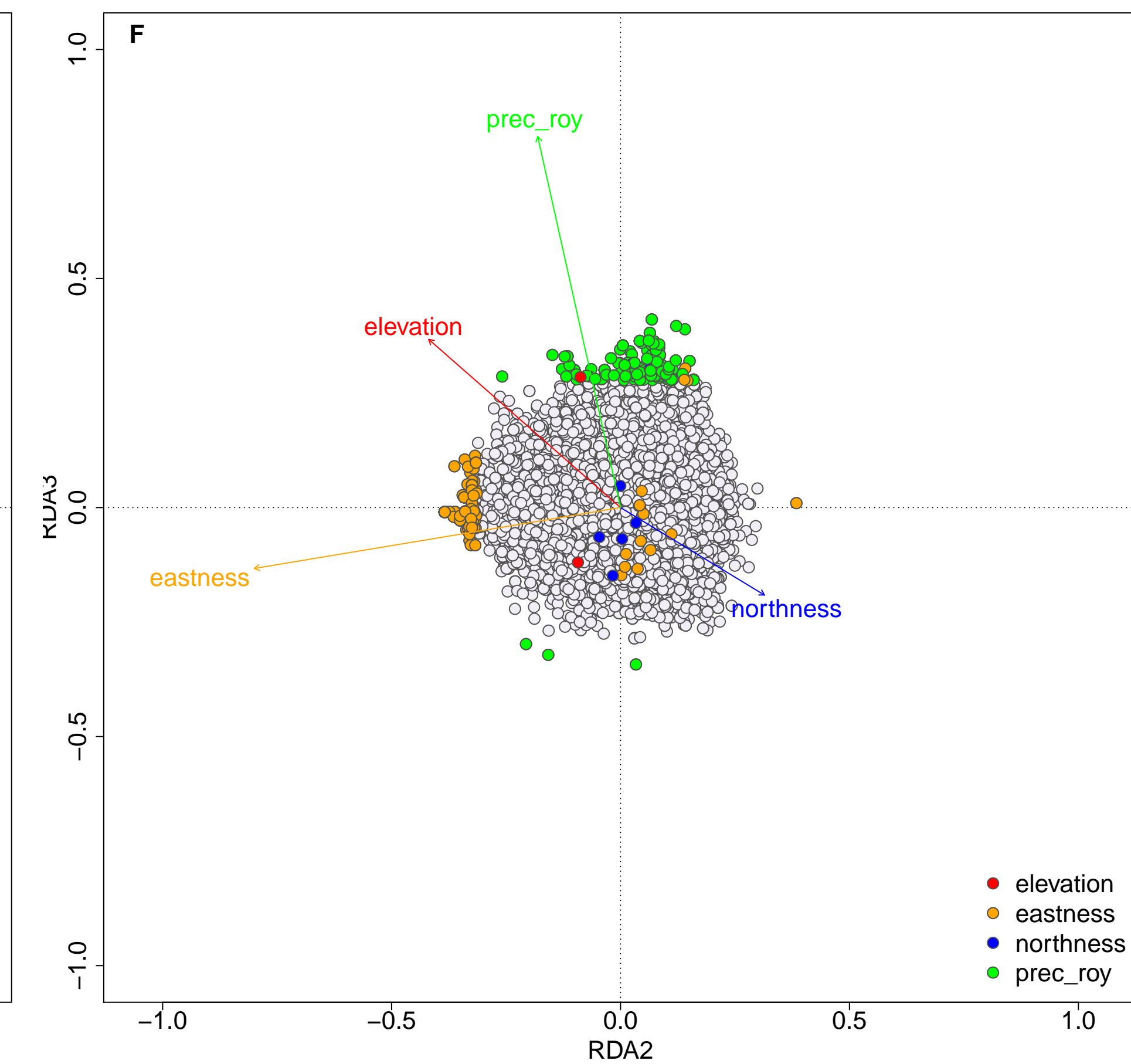

Supplement: Supplementary file 19 — Additional file 19: Figure S12. Triplots of the first three significant axis of the Redundancy Analysis (RDA) show variants (grey open circles) and environmental variables (colored arrows; eastness, elevation, northness and precipitation outside of the vegetation period (September–May; prec_roy)) for Achillea clusiana (A–C) and for Campanula pulla (D–F), respectively. Variants are colored depending on which environmental variable they significantly correlate with. [file 12870_2023_4187_MOESM19_ESM.pdf]
