# Supplementary material for: Genome-environment associations along elevation gradients in two snowbed species of the North-Eastern Calcareous Alps
Source: BMC Plant Biol. 2023 Apr 19;23:203. doi: 10.1186/s12870-023-04187-x (PMC10114330; doi:10.1186/s12870-023-04187-x)
Supplement: Supplementary file 18 — Additional file 18: Figure S11. Allele frequencies for quality filtered variants for A) Achillea clusiana populations and B) Campanula pulla populations. Allele frequency range was split into ten bins of bin size ten with stepsize ten. Population names consist of species (A for A. clusiana, C for C. pulla), mountain (K for Admonter Kaibling, H for Hochschwab, and S for Schneeberg) and elevation (lower L, medium M, and upper U). [file 12870_2023_4187_MOESM18_ESM.pdf]

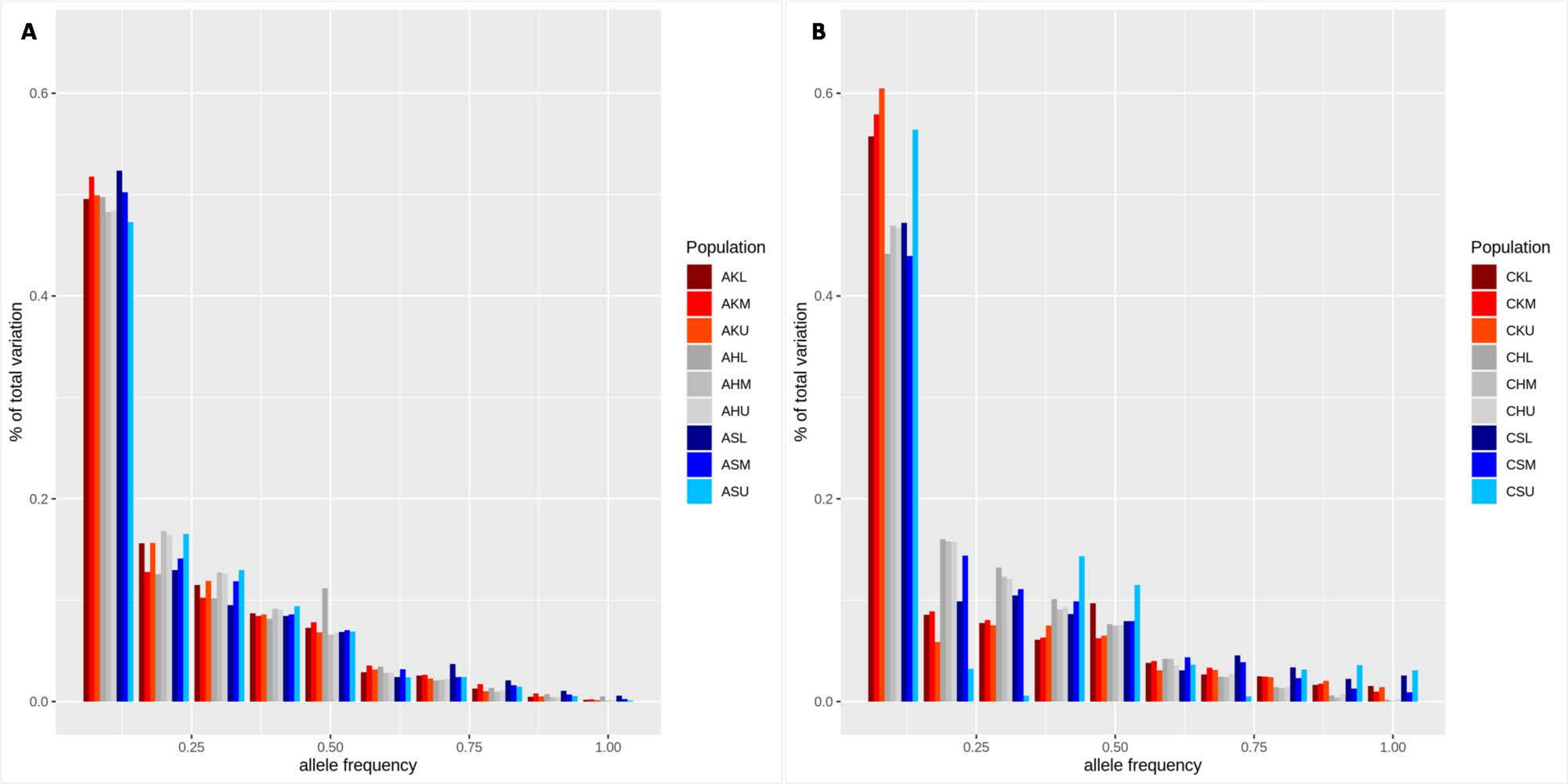
A
B
% of total variation
0.0
0.2
0.4
0.6
allele frequency
0.25
0.50
0.75
1.00
Population
AKL
AKM
AKU
AHL
AHM
AHU
ASL
ASM
ASU
CKL
CKM
CKU
CHL
CHM
CHU
CSL
CSM
CSU
